# Application of grouping and read-across for the evaluation of parabens of different chain lengths with a particular focus on endocrine properties

**DOI:** 10.1007/s00204-020-02967-0

**Published:** 2021-01-18

**Authors:** Susann Fayyaz, Reinhard Kreiling, Ursula G. Sauer

**Affiliations:** 1 Clariant Produkte (Deutschland) GmbH, Am Unisyspark 1, 65843 Sulzbach, Germany; 2 Scientific Consultancy-Animal Welfare, Neubiberg, Germany

**Keywords:** Linear *n*-alkyl parabens, Repeated-dose toxicity, Endocrine disruptor, Developmental and reproductive toxicity (DART), Grouping and read-across, Risk assessment, Registration, Evaluation, Authorisation and Restriction of Chemicals (REACH)

## Abstract

This article presents the outcomes of higher-tier repeated-dose toxicity studies and developmental and reproductive toxicity (DART) studies using Wistar rats requested for methyl paraben and propyl paraben under the European Union chemicals legislation. All studies revealed no-observed adverse effects (NOAELs) at 1000 mg/kg body weight/day. These findings (absence of effects) were then used to interpolate the hazard profile for ethyl paraben, further considering available data for butyl paraben. The underlying read-across hypothesis (all shorter-chained linear *n*-alkyl parabens are a ‘category’ based on very high structural similarity and are transformed to a common compound) was confirmed by similarity calculations and comparative in vivo toxicokinetics screening studies for methyl paraben, ethyl paraben, propyl paraben and butyl paraben. All four parabens were rapidly taken up systemically following oral gavage administration to rats, metabolised to *p*-hydroxybenzoic acid, and rapidly eliminated (parabens within one hour; *p*-hydroxybenzoic acid within 4–8 h). Accordingly, for ethyl paraben, the NOAELs for repeated-dose toxicity and DART were interpolated to be 1000 mg/kg body weight/day. Finally, all evidence was evaluated to address concerns expressed in the literature that parabens might be endocrine disruptors. This evaluation showed that the higher-tier studies do not provide any indication for any endocrine disrupting property. This is the first time that a comprehensive dataset from higher-tier in vivo studies following internationally agreed test protocols has become available for shorter-chained linear *n*-alkyl parabens. Consistently, the dataset shows that these parabens are devoid of repeated-dose toxicity and do not possess any DART or endocrine disrupting properties.

**Supplementary Information:**

The online version contains supplementary material available at 10.1007/s00204-020-02967-0.

## Introduction

### Background

Parabens are alkyl or aryl esters of *p*‐hydroxybenzoic acid, an essential and ubiquitous plant constituent in cereals, fruit, vegetables and spices thought to act as common natural defence against bacterial and fungal infections (Aubert et al. 2012). Just as the parent compound, parabens exhibit a broad variety of antibacterial and antifungal effects. Further, parabens are chemically stable, non-volatile and odourless; they are generally of very low systemic toxicity upon short- or long-term exposure, and they lack allergic potential (Hafeez and Maibach 2013; Soni et al. 2005; Fransway et al. 2019a, b). Parabens with different *n*-alkyl chain lengths ranging from methyl paraben, ethyl paraben, propyl paraben to butyl paraben have been widely and safely used for many decades as preservatives in a variety of cosmetics, foods, beverages and pharmaceuticals (Soni et al. 2001, 2002, 2005).

In the European Union, the safe manufacture and occupational handling of parabens, as chemical substances, is regulated by *Regulation (EC) No 1907/2006 concerning the Registration, Evaluation, Authorisation and Restriction of Chemicals* (REACH; EP and Council 2006). The REACH registration procedure includes preparation of a dossier presenting all relevant data to identify the substance and to assess any potential risks related to it. As per REACH Annexes VII–X, the extent of information to be included in the dossiers depends on the annual tonnage at which the given substance is manufactured or imported (EP and Council 2006). This set of Annexes contains not only increasingly comprehensive standard information requirements, but also specific rules for their adaptation. The applicable REACH Annexes differ between the parabens since methyl paraben is manufactured or imported at > 1000 tonnes per year (tpy), ethyl paraben and propyl paraben each at > 100 tpy, and butyl paraben at 1–10 tpy (Table 1).

**Table 1:**
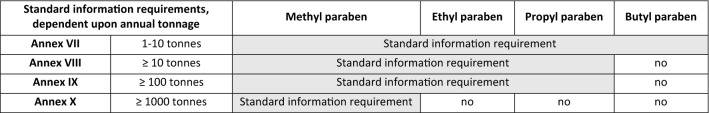
Standard information requirements for methyl paraben, ethyl paraben, propyl paraben and butyl paraben as per REACH Annexes VII-X (EP and Council, 2006)

Following the respective REACH information requirements and subsequent requests by the European Chemicals Agency (ECHA 2017a, 2018), higher-tier (i.e. REACH Annex IX and X) studies have been requested and conducted for methyl paraben and propyl paraben, including:

- A rat oral 90-day repeated-dose toxicity study (Organisation for Economic Co-operation and Development (OECD) Test Guideline (TG) 408);
- A prenatal developmental toxicity study (OECD TG 414), which was requested for propyl paraben, but was already available for methyl paraben (Food and Drug Research Laboratories 1972).
- A reproductive toxicity screening study possibly combined with a 28-day repeated-dose toxicity study (OECD TG 421/422); and
- An extended one-generation reproduction toxicity study (OECD TG 443).

For ethyl paraben, data from these studies are currently unavailable. European Union (EU) legislation mandates that animal testing must be avoided as far as possible: Article 25(1) of the REACH Regulation requires that testing on vertebrate animals shall be undertaken only as a last resort. Further, the 3Rs principle to replace, reduce and refine animal testing (Russell and Burch 1959) has been implemented in *Directive 2010/63/EU on the protection of animals used for scientific purposes* (EP and Council 2010).

Ethyl paraben (C_9_H_10_O_3_) only differs from methyl paraben (C_8_H_8_O_3_) and propyl paraben (C_10_H_12_O_3_) by having one CH_2_-unit more in the linear *n*-alkyl moiety than methyl paraben and one CH_2_-unit less than propyl paraben. Due to the high structural similarity between these three substances, grouping and read-across appears a practicable approach to minimise animal testing for the hazard assessment of ethyl paraben.

Against this background, this research article presents and discusses the outcomes of the higher-tier studies for methyl paraben and propyl paraben, requested and conducted under the REACH Regulation. These findings are then used for grouping and read-across following internationally agreed scientific principles (OECD 2014; ECHA 2017b) to interpolate the corresponding hazard profile for ethyl paraben. As supporting evidence, the findings from in vivo toxicokinetics screening studies for methyl paraben, ethyl paraben, propyl paraben and butyl paraben are presented and discussed, further considering available data for the acute toxicity, local toxicity and genotoxicity endpoints.

### Grouping and read-across as practicable approach to minimise animal testing

*Grouping* is defined as the general approach for considering more than one chemical at the same time during hazard and risk assessment (OECD 2014). The *category approach* is employed between several substances that are grouped together based on defined structural similarity for one or more (toxicological or other) properties (OECD 2014; ECHA 2017b). *Read-across* is defined as a technique for predicting endpoint information for the *target substance* by using available data for the same endpoint from the *source substance(s)* (OECD 2014; ECHA 2017b). Read-across can be conducted by *interpolation*, i.e. by estimating a value for a category member using measured values from other members on both sides of that member within the defined category spectrum (ECHA 2008; OECD 2014). By contrast, *extrapolation* refers to the estimation of a value for a member that is near or at the category boundary using measured values from internal category members (ECHA 2008; OECD 2014).

Category approaches have been successfully used to minimise animal testing, e.g. in the OECD High Production Volume Programme (OECD 2014) as well as in the U.S. High Production Volume Challenge Program (Stanton and Kruszewski 2016), and also, in some cases, under the REACH Regulation (Ball et al. 2016). Examples of accepted categories comprise inter alia aliphatic acids, alpha-olefins, long-chain alcohols, linear alkylbenzene sulfonates and primary alkyl amines (OECD 2014; see also https://hpvchemicals.oecd.org/ui/ChemGroup.aspx).

ECHA (2008,2013,2017b) has published guidance to support the harmonisation of grouping and read-across under the REACH Regulation (EP and Council 2006). Similarity, as pivotal aspect for grouping substances into categories, may be established based upon (amongst other aspects) the following:

- “Common functional group(s) (e.g. aldehyde, epoxide, ester, specific metal ion)”;
- “An incremental and constant change across the category (e.g. a chain-length category)”;
- “The likelihood of common precursors and/or common breakdown products via physical or biological processes, which result in structurally similar chemicals (e.g. the metabolic pathway approach of examining related chemicals such as acid/ester/salt)” [ECHA (2008); same wordings in OECD (2014)].

**Fig. 1:**
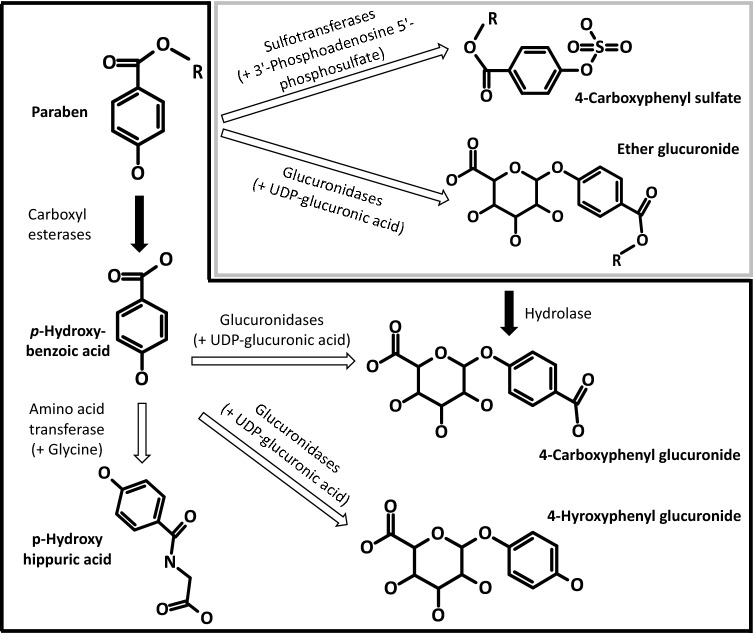
Common pathways of metabolism of methyl paraben, ethyl paraben, propyl paraben, and butyl paraben Methyl paraben: R = –CH_3_; ethyl paraben: R = CH_2_–CH_3_; propyl paraben: R = CH_2_–CH_2_–CH_3_; butyl paraben: R = CH_2_–CH_2_–CH_2_–CH_3_. Black arrows relate to phase I enzymes and white arrows to phase II enzymes (UGT: Uridine 5′-diphospho-glucuronosyltransferase). Parent compound and metabolites are presented in bold font; enzymes and their further substrates in normal font. The major metabolic pathway for parabens (metabolism back to *p*-hydroxybenzoic acid followed by conjugation) is highlighted by the black frame; the subordinate pathway (direct sulfation/glucuronidation of the parent compound) by the grey frame

Applying these principles, linear *n*-alkyl parabens are considered a *‘*chemical category’ (i.e. esters of *p*‐hydroxybenzoic acid) based on structural and functional similarity, and a ‘chain-length category’ as they deviate only in the number of CH_2_-units of the alkyl moiety. Further and importantly, they are considered a ‘metabolic pathway category’ since they all follow the same metabolic pathway and are thus regarded biologically equivalent (Fig. 1). All parabens are equally and readily metabolised back to *p*-hydroxybenzoic acid by esterases in different tissues, thereafter conjugated with sulfate, glucuronide or glycine, and then rapidly excreted in the urine; subordinate metabolic pathways are sulfation and glucuronidation of the parent compounds, which are then also rapidly excreted (Soni et al. 2005; Abbas et al. 2010; Aubert et al. 2012; Ozaki et al. 2013).

The entirety of linear *n*-alkyl parabens also includes parabens with longer *n*-alkyl moieties (pentyl paraben, hexyl paraben, heptyl paraben, etc.). However, these parabens do not have economic relevance and have not been registered under the REACH Regulation. Therefore, data for these longer-chained linear *n*-alkyl parabens are generally scant, and they are unavailable on the ECHA dissemination portal; https://echa.europa.eu/. Indeed, usage of pentyl paraben in cosmetic products was banned in the European Union in 2014, not on account of specific health concerns, but since “limited or no information was submitted by industry for the safety evaluation” (Commission 2014).

Focus of the present article is on the shorter-chained linear *n*-alkyl parabens and an interpolation of missing data for ethyl paraben (as target substance) using measured values from other members on both sides of that member within the defined category spectrum (i.e. methyl paraben and propyl paraben as source substances) as requested in ECHA (2008) and OECD (2014).

ECHA (2008) clearly denotes that interpolation is preferred to extrapolation and that the extrapolation of missing data requires special considerations. For this reason, the further application of read-across to extrapolate missing data for butyl paraben (C_11_H_14_O_3_; i.e. whose linear *n*-alkyl chain has one CH_2_-unit more than propyl paraben) is not a focus of this article and only briefly addressed in the discussion (see “Tentative extrapolation of missing data for butyl paraben”). Notably, for butyl paraben, higher-tier studies are not standard information requirements under REACH on account of its lower production volume (Table 1).

### Follow-up of concern for endocrine disrupting potential of parabens

The performance of the extended one-generation reproductive toxicity studies for methyl paraben and propyl paraben [and including the developmental immunotoxicity (DIT) and developmental neurotoxicity (DNT) cohorts] was specifically requested by ECHA (2017a,^2018^) based on concern triggers relating to reproductive toxicity and endocrine activity. Hence, the present article pursues a second aim, i.e. to follow up the concern for endocrine disrupting potential of shorter-chained linear *n*-alkyl parabens.

As defined by the World Health Organisation International Programme on Chemical Safety (WHO IPCS 2002), an endocrine disruptor is an “exogenous substance or mixture that alters function(s) of the endocrine system and consequently causes adverse health effects in an intact organism, or its progeny, or (sub)populations”. The OECD (2012,2018) has published the *OECD Conceptual Framework (CF) for Testing and Assessment of Endocrine Disrupting Properties* that presents five levels of information, assays and studies (mostly OECD TGs) that are useful for the determination of endocrine disruption (Table 2).

**Table 2:** Overview of the OECD Conceptual Framework for Testing and Assessment of Endocrine Disruptors and the corresponding OECD TGs for human health hazard assessment (adapted from OECD (2012) and the respective OECD TGs)

OECD CF level	Description	Examples for relevant Information/relevant mammalian toxicity test methods	For Level 3 assays and Level 4 and Level 5 studies: details from corresponding OECD TG
Level 1	Existing data and non-test information	Physical and chemical properties	Please refer to the OECD (2018) Guidance Document No. 150 for further details on the test protocols
		All available (eco)toxicological data from standardised or non-standardised tests	
		Read-across, chemical categories, in silico and ADME model predictions	
Level 2	In vitro assays providing data about selected endocrine mechanisms/pathways	Oestrogen or androgen receptor binding affinity	
		OECD TG 455–457: oestrogen receptor transactivation	
		MCF-7 cell proliferation assays (oestrogen receptor antagonist/agonist)	
Level 3	In vivo assays providing data about selected endocrine mechanisms/pathways; for mammalian toxicology	OECD TG 440: uterotrophic assay in rodents	Test substance administered orally or subcutaneously for three consecutive days to either immature or ovariectomised females For oestrogen agonists, statistically significant increase in mean uterine weight of the treated animal groups relative to vehicle group
		OECD TG 441: Hershberger assay in rats	Test substance administered orally or subcutaneously to castrate-peripubertal male rats for 10 consecutive days (together with reference androgen agonist for testing for antiandrogens) Significant weight change in two of five androgen-dependent tissues
Level 4	In vivo studies providing data on adverse effects on endocrine relevant endpoints	OECD TG 407 and 408: repeated dose 28-day and 90-day toxicity studies	Test substance administered orally and daily to rats for 28/90 days Study results include clinical examination, gross necropsy and histopathology; number of endocrine-related measurements, particularly relevant to thyroid function, have been added in 2018
		OECD TG 414: prenatal developmental toxicity study	Test substance administered to pregnant dams from implantation to shortly before delivery Study results include clinical examination, examinations of uterine contents and of foetuses for soft tissue and skeletal changes; a number of endocrine-related measurements (e.g. serum T3, T4, TSH levels in dams/foetuses) were added in 2018
		OECD TG 421; if enhanced: reproductive screening test	Similar to OECD TG 422; see below
		OECD TG 422; if enhanced: combined 28-day/reproductive screening assay	Test substance administered orally and daily to males for ≥ 4 weeks and females throughout study (approx. 63 days) Study results include clinical observations, oestrous cycle monitoring, offspring parameters observation/measurement, thyroid hormone measurement, gross necropsy and histopathology; measurements of anogenital distance and male nipple retention in pups, and thyroid examination, were added in 2016
Level 5	In vivo studies providing more comprehensive data on adverse effects on endocrine relevant endpoints over more extensive parts of the life cycle of the organism	OECD TG 443: extended one-generation reproduction toxicity study	Sexually-mature male and female rodents (parental (P_0_) generation) exposed to test substance from 2 weeks before mating and all through mating, gestation and weaning of pups (F_1_ generation); at weaning, pups are assigned to cohorts for reproductive/developmental toxicity (cohort 1), DNT (cohort 2) and DIT (cohort 3) testing. F_1_ offspring are treated from weaning to adulthood; part of cohort 1 (cohort 1B) may be extended to F_2_ generation (procedures for F_1_ animals similar to those for the P_0_ animals) All animals: clinical observations and pathology examinations; specifically: integrity and performance of male and female reproductive systems and health, growth, development and function of offspring
		OECD TG 416; version of 22 January 2001: two-generation reproduction toxicity study	Comparable to OECD TG 443, but mandatory 2nd generation; but not relevant for the present research article

*ADME* Absorption, distribution, metabolism, elimination, *CF* Conceptual framework, *DIT* Developmental immunotoxicity, *DNT* Developmental neurotoxicity, *GD* Gestational day, *PND* Postnatal day, *T3* Tri-iodothyronine, *T4* Thyroxine, *TG* Test guideline, *TSH* Thyroid stimulating hormone

The concerns relating to reproductive toxicity and endocrine activity expressed by ECHA (2017a,^2018^) were predominantly associated with results from in vitro and in vivo screening assays that correspond to OECD CF Level 2 and Level 3 assays. These findings mainly relate to in vitro oestrogen receptor transactivation or in vivo uterus weight increase (Routledge et al. 1998; Blair et al. 2000; Byford et al. 2002; Cashman and Warshaw 2005; Golden et al. 2005; Brand et al. 2017; Nowak et al. 2018). The ability of parabens to transactivate oestrogen receptors in in vitro OECD CF Level 2 assays increases with alkyl chain length. From amongst the shorter-chained linear *n*-alkyl parabens, methyl paraben elicits the least and butyl paraben the most activation (Byford et al. 2002).

By contrast, across the entirety of linear *n*-alkyl parabens, a U-shaped association between chain length and in vitro interaction with oestrogen receptors is observable: amongst 12 parabens with linear *n*-alkyl chains ranging in length from C_1_ to C_12_, heptyl paraben (C_14_H_20_O_3_, i.e. C_7_-alkyl chain) and pentyl paraben (C_12_H_16_O_3_, i.e. C_5_-alkyl chain) showed the highest potency in activating human oestrogen receptors α and β; at 10^–7^ M and 10^–8^ M, respectively (Watanabe et al. 2013). The potency of oestrogen receptor activation decreased in a stepwise manner (and the lowest concentration inducing receptor effects increased) as the alkyl chain was shortened to C_1_ (methyl paraben) or lengthened to C_12_ (dodecyl paraben) (Watanabe et al. 2013). Importantly, all receptor effects (also those of the most active heptyl paraben and pentyl paraben) only occurred at concentrations that were many orders of magnitude higher than that of the natural oestrogen 17β-estradiol (2.5 × 10^–12^) (Watanabe et al. 2013), i.e. the commonly used positive control in in vitro oestrogen receptor transactivation studies (Soni et al. 2005; Watanabe et al. 2013; US EPA 2015). These observations further support the decision to focus only on the shorter-chained linear *n*-alkyl parabens in the category approach to interpolate missing data for ethyl paraben.

Borgert et al. (2018) have suggested a human-relevant potency threshold for oestrogen receptor α agonism of 10^–4^ relative to the potency of 17β-estradiol as minimum level of mechanistic potency necessary for a chemical to be able to act via this mode-of-action in humans. Following this threshold, none of the in vitro oestrogen receptor activation potencies of the linear *n*-alkyl parabens (Watanabe et al. 2013) are sufficiently high to act via an oestrogenic mode-of-action in humans.

Assessments of shorter-chained linear *n*-alkyl parabens in OECD CF Level 3 rodent uterotrophic assays yielded equivocal results (Routledge et al. 1998; Soni et al. 2001, 2002; CIR 2008, 2019; Ohta et al. 2012).

A variety of mostly non-TG-conform studies have investigated whether parabens have the potential to affect the reproductive system as important target organ system for endocrine disruptors. Conflicting reports are available on effects of parabens on the male or female reproductive system (see “Evaluation of linear *n*-alkyl parabens under product-specific EU legislation and in the scientific literature”). 

As the U.S.-based Cosmetic Ingredients Review (CIR) Expert Panel denoted:

1. *“Many of these reports are irrelevant to the routes of exposure associated with intended cosmetic use, or otherwise did not account for the extensive metabolism of parabens (to metabolites with no known developmental and reproductive toxicity activity)*;
2. *are the result of poorly designed studies*; and
3. *were not verified by other methods*” (CIR 2019).

The higher-tier studies requested under REACH Annexes IX–X for methyl paraben and propyl paraben correspond to Level 4 and Level 5 studies of the OECD CF (OECD 2012). While OECD CF Level 2 and Level 3 assays inform on a substance’s potential to exhibit endocrine activity (e.g. in vitro oestrogen receptor transactivation and in vivo uterus weight increase), only OECD CF Level 4 and Level 5 studies also inform on a substance’s potential to elicit adverse effects in an intact organism as a consequence of the endocrine activity. Such adversity is an indispensable prerequisite to meet the definition of an endocrine disruptor (WHO IPCS 2002). Therefore, for the first time, the findings presented in this article enable a comprehensive evaluation of the endocrine disrupting potential of shorter-chained linear *n*-alkyl parabens according to all five levels of the OECD CF (Table 2).

Since the OECD CF Level 4 and Level 5 studies served regulatory purposes, they were performed following internationally agreed, standardised test protocols (i.e. OECD TGs). Thereby, the relevance, reliability and repeatability of findings is ensured, and it is ascertained that animal group sizes are adequate for the statistical analysis of findings. Adherence to OECD TGs also facilitates the mutual acceptance of data on an international level (OECD 2020). Study relevance was further enhanced by conducting all studies in full compliance with the principles of Good Laboratory Practice (GLP; OECD 1998).

Taken together, all findings presented in this article will be used in a weight-of-evidence approach to derive an overall conclusion on whether, or not, shorter-chained linear *n*-alkyl parabens might exhibit endocrine disrupting properties. Focus is on methyl paraben, ethyl paraben, and propyl paraben, while briefly referring to butyl paraben. Further, the discussion briefly addresses the sodium salts of methyl paraben, ethyl paraben, and propyl paraben. Notably, all evaluations are restricted to the human health endpoints. Ecological endpoints, while being relevant for a comprehensive hazard and risk assessment, are out of scope of this article. Also, only the shorter-chained linear *n*-alkyl parabens are considered, but neither longer-chained linear *n*-alkyl parabens (pentyl paraben, hexyl paraben, etc.), *iso*-alkyl parabens (e.g. *iso-*butyl paraben), or aryl parabens (e.g. benzyl paraben).

## Materials and methods

### Test items and test item preparation

Methyl paraben, ethyl paraben, propyl paraben and butyl paraben were obtained from Clariant Produkte (Deutschland) GmbH and certified analytically regarding chemical identity and purity. Generally, these parabens have very similar physico-chemical properties. They are slightly soluble or insoluble crystalline solids, with melting points above 65 °C, acid dissociation constants of approx. 8.4 and octanol–water partition coefficients ranging between approx. 2 and 3.5 (Table 3).

**Table 3:**
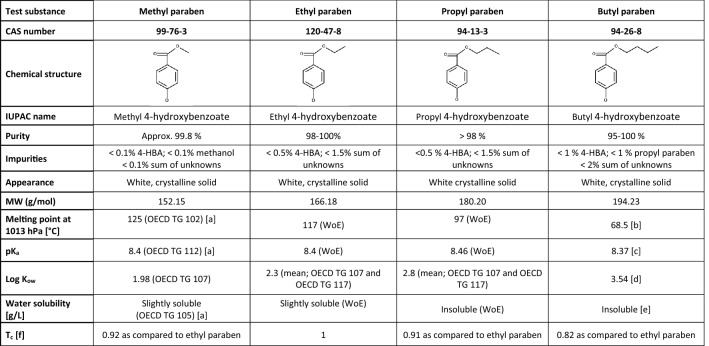
Physical and chemical properties of methyl paraben, ethyl paraben, propyl paraben, and butyl paraben

This table contains data that are freely available on the ECHA dissemination portal (https://echa.europa.eu/). Purity of all test items was confirmed via Certificate of Analysis by the producer’s Quality Assurance Unit

*4-HBA* 4-Hydroxybenzoate, *CAS* Chemical Abstract Service, *IUPAC* International Union of Pure and Applied Chemistry, L*og K*_*ow*_ Octanol–water partition coefficient, *MW* Molecular weight, *PK*_*a*_ (Negative base-10 logarithm of) acid dissociation constant, *T*_*c*_ Tanimoto similarity coefficient, *WoE* Weight-of-evidence

References: [a] Study performed or commissioned by Clariant Produkte (Deutschland) GmbH; [b] Lide (2005); [c] Dymicky and Huhtanen (1979), cited in CIR (2008); [d] Hansch et al. (1995); [e] Yalkowsky and He (2003). [f] The Tanimoto similarity coefficient (*T*_c_) was calculated using the Open Babel open source chemistry toolbox, version 2.4.1 (O’Boyle et al. 2011). A *T*_c_ > 0.85 is assessed as indicating high similarity and a *T*_c_ > 0.7 as indicating similarity (see Sect. 2.3 for further details)

The test items were prepared with 1% aqueous hydroxyethyl-cellulose (Sigma Aldrich, Germany). This vehicle was also administered (via gavage) to the control groups. Test items were prepared once for the toxicokinetics studies (since it includes single substance administration) and once every four days for the higher-tier (repeated-dose) studies assessing methyl paraben and propyl paraben (since this interval was identified as suitable during preliminary stability studies).

### In vivo studies

All in vivo studies were conducted at BSL BIOSERVICE in Planegg, Germany. BSL BIOSERVICE has full accreditation from the Association for the Assessment and Accreditation of Laboratory Animal Care (AAALAC) International. It has been certified in accordance with GLP and Good Manufacturing Practice (GMP), and it has further been accredited with DIN EN ISO 17025 for biocompatibility testing of medical devices. In accordance with the German Animal Protection Law, all studies were subjected to the ethical review process and authorised by the Animal Welfare Administration of the Government of Upper Bavaria (Germany).

Healthy and specific pathogen-free male and female (non-pregnant and nulliparous) Wistar Crl: WI(Han) rats were obtained from Charles River Laboratories (Germany). The animals were housed in an air-conditioned room (temperature: 22 ± 3 °C; relative humidity: 55 ± 10%; artificial light, light–dark cycle: 12 h–12 h; air change: 10 times/h). Housing was in groups of 5 animals/sex/cage in individually ventilated Type IV polysulphone cages on Altromin saw fibre bedding with free access to both Altromin 1324 maintenance diet for rats and mice and tap water. Upon arrival at BSL BIOSERVICE, the animals underwent an acclimatisation period of at least 5 days, and they were approximately 15–16 weeks old at the onset of the treatment period.

All in vivo studies were conducted as oral gavage studies, selecting the oral route as most appropriate route of administration to reflect the worst-case exposure scenario. Both methyl paraben and propyl paraben were assessed using identical test protocols, i.e. the rodent 90-day repeated-dose toxicity study (OECD TG 408); the reproductive toxicity screening study combined with a 28-day repeated dose toxicity study (OECD TG 422); and the extended one-generation reproductive toxicity study (OECD TG 443). The OECD TG 443 included mating of first generation (F_1_) offspring to produce a second generation (F_2_), and the DIT and DNT cohorts. Propyl paraben was additionally tested in the prenatal developmental toxicity study (OECD TG 414), whereas a historical OECD TG 414-like study was available for methyl paraben (Food and Drug Research Laboratories 1972) (Table 4).

**Table 4:** Overview of in vivo higher-tier studies performed for the assessment of methyl paraben and propyl paraben

Test method	OECD TG	Test item	Number of male and female animals per group	Dose groups (mg/kg bw/day)
90-day repeated dose oral toxicity study	OECD TG 408 (version of 2018)	Methyl paraben	80 animals (40 m and 40 f) included in study (10 m and 10 f/group); additionally, 20 animals (5 m and 5 f/group): recovery animals (control and high dose groups)	0, 100, 300, 1000
		Propyl paraben		
Prenatal developmental toxicity study [a]	OECD TG 414 (version of 2001)	Propyl paraben	156 P_0_ (52 m and 104 f) + 965 pups	0, 100, 300, 1000
Reproductive toxicity screening study possibly combined with 28-day repeated dose toxicity study	OECD TG 422 (version of 2016)	Methyl paraben	80 P_0_ (40 m and 40 f) + 462 pups	0, 500, 1000
	OECD TG 421 (version of 2016)	Propyl paraben	50 P_0_ (25 m and 25 f) included in study (5 m and 5 f in control group; 10 m and 10 f/dose group) + 276 pups [b]	
Extended one-generation reproductive toxicity study	OECD TG 443 (version of 2018)	Methyl paraben	220 P_0_ (110 m and 110 f) included in study (25 m and 25 f/group in low- and mid-dose groups; 30 m and 30 f/group in control and high-dose groups); reserve animals (2 per sex); approx. 1750 pups	0, 100, 300, 1000
		Propyl paraben	220 P_0_ (110 m and 110 f) included in study (25 m and 25 f/group in low- and mid-dose groups; 30 m and 30 f/group in control and high-dose groups); reserve animals (2 per sex); approx. 1750 pups	

In all studies, Wistar rats were used. Test materials were prepared in 1% hydroxyethyl-cellulose. Oral gavage application once daily for 7 days/week, at a volume of 5 mL/kg bw. The respective vehicle control groups received 1% hydroxyethyl-cellulose. All dosages refer to nominal doses. All studies have a GLP certificate with the exception of the OECD TG 421 for propyl paraben that was conducted following the principles of GLP, but without GLP certification since it served as dose range-finding study (see [a]). All studies were requested under the REACH Regulation; for complete test protocols, see: http://www.oecd.org/chemicalsafety/testing/oecdguidelinesforthetestingofchemicals.htm

*bw* Body weight, *f* Female, *F*_*1*_ First generation offspring, *m* Male, *NOAEL* No-observed adverse effect level, *P*_*0*_ Parental animals

[a] A historical OECD TG 414-like prenatal developmental toxicity study was available for methyl paraben (Food and Drug Research Laboratories 1972)

[b] Conducted as oral gavage dose range-finding study for the OECD TG 443 with reduced animal numbers since an OECD TG 422 rat feeding study (Harlan 2012) was already available (but not suitable as dose range finding study due to the difference in the application method)

Notably, for methyl paraben, the set of higher-tier tests includes an OECD TG 422 (i.e. reproductive toxicity screening study combined with a 28-day repeated dose toxicity study) conducted as oral gavage study. This OECD TG 422 also served as dose range finding study for assessing methyl paraben in the subsequent OECD TG 443. For propyl paraben, a historical OECD TG 422 study was available (Harlan 2012). Since that study had been conducted as feeding study, it was considered unsuitable as range finding study for the OECD TG 443 that was planned as oral gavage study. Therefore, for propyl paraben, the dose range finding for the OECD TG 443 [that was also requested by ECHA (2017a)] was undertaken as screening study following the OECD TG 421 test protocol with reduced numbers of animals to comply with the legally mandated 3Rs principle (EP and Council 2010).

Further, methyl paraben, ethyl paraben, propyl paraben, and butyl paraben were submitted to in vivo toxicokinetic screening studies. These studies were conducted similarly to OECD TG 417 applying 500 and 1000 mg/kg body weight (bw) test item via oral gavage to 10 males and 10 females per dose group. Focus of the toxicokinetic evaluation was on the measurement of the serum concentrations of the parent compounds and their (common) major metabolite *p*-hydroxybenzoic acid by inductively coupled plasma - mass spectrometry (ICP-MS) in order to assess the systemic uptake and elimination of the parent compounds and this major metabolite [see also ECHA (2017a)]. The limit of quantification was determined as 10 ng/mL for all parabens, and as 130 ng/mL for *p*-hydroxybenzoic acid. Notably, these comparative toxicokinetic studies were conducted as screening tests with the major aims to first check whether significant differences in the systemic uptake behaviour exist between the investigated parabens, and second to rule out that either the parent compounds or *p*-hydroxybenzoic acid would be present in the blood circulation for sufficiently long periods of time to elicit adverse effects (further taking into account that *p*-hydroxybenzoic acid is not toxic; see Subsection AE C.4 in “Common assessment elements (AEs) for category approaches”). On account of these major aims, investigations of the faeces and urine were not included in the toxicokinetics screening studies.

All in vivo studies were required under the REACH Regulation (EP and Council 2006).

### Application of read-across to fill data gaps for ethyl paraben

Read-across to interpolate missing data for the target substance ethyl paraben (C_9_H_10_O_3_) by using the data available for the source substances methyl paraben (C_8_H_8_O_3_) and propyl paraben (C_10_H_12_O_3_) was performed in accordance with Scenario 5 described in the ECHA (2017b) *Read-Across Assessment Framework* (RAAF):

- The category approach was followed.
- The read-across hypothesis was based on *“(bio)transformation to common compounds*”.
- It was hypothesised that *“no relevant differences in predicted properties are observed for several source substances*” (ECHA 2017b). (The predicted properties relate to adversity and not endocrine activity.)

The evaluation of the read-across followed the specific assessment elements (AEs) described for Scenario 5 in the ECHA RAAF (see “Ethyl paraben: Interpolation from methyl paraben and propyl paraben”). The strength of evidence for the respective AEs was scored by the assessment options indicated in the RAAF ranging from Score 5 (acceptable with high confidence) to Score 1 (not acceptable) (ECHA 2017b).

To further support the read-across, historical data for acute toxicity, eye and skin irritation, skin sensitisation, and genotoxicity were collated for methyl paraben, ethyl paraben, propyl paraben, and butyl paraben (Table 5).

**Table 5:** Historical acute toxicity, local toxicity and genotoxicity data for methyl paraben, ethyl paraben, propyl paraben, and butyl paraben

Endpoint	Methyl paraben	Ethyl paraben	Propyl paraben	Butyl paraben
*Acute oral toxicity studies using rats: LD*_*50*_* [mg/kg bw] (in brackets: OECD TG and date of study)*				
Acute toxicity	2100 (OECD 401; 1974)	> 3100 (OECD 401; 1982)	> 5000 (OECD 401; 1982)	> 2000 (read across; OECD 423; 2018) [a, b]
*Local toxicity studies using rats: GHS/CLP classification (in brackets: OECD TG and date of study)*				
Skin irritation/skin corrosion	Not irritating (modif. Draize skin irritation test; 1976)	Not irritating (OECD 404; 1983)	Not irritating (read-across)	Irritating (read-across; OECD 439; 2016) [a]
Eye irritation/eye corrosion	Not irritating (modif. Draize eye irritation test; 1976)	Not irritating (OECD 405; 1983)	Not irritating [c] (OECD 437 and 405; 2012)	Ser. eye damage (read-across; OECD 437; 2016) [a]
Skin sensitisation	Not sensitising (equiv. OECD 406; 1980)	Not sensitising (equiv. OECD 406; 1981)	Not sensitising (OECD 406 and 429; 1992)	Not sensitising (read-across; OECD 429; 2016) [a]
*In vitro and in vivo genotoxicity studies: GHS/CLP classification (in brackets: OECD TG and date of study)*				
Mutagenicity in bacteria	Not mutagenic (OECD 471; 1982, 1991)	Not mutagenic [c] (OECD 471; 2012)	Not mutagenic [c] (OECD 471; 2018)	Not mutagenic (read-across; OECD 471; 2016) [a]
Mutagenicity in mammalian cells	Not mutagenic [c] (OECD 476; 2019)	Not available	Not mutagenic [c] (OECD 476; 2012)	Not available
Cytogenicity in mammalian cells	Clastogenic with metabolic activation (OECD 473; 1987) [d]	Not available	Not clastogenic [c] (OECD 487; 2018)	
Genetic toxicity in vivo (rats)	Not clastogenic (OECD 478; 1974) [d]	Not clastogenic (read-across from MP)	Not clastogenic (read-across from MP)	

This table contains data that are freely available on the ECHA dissemination portal (https://echa.europa.eu/). Years related to the date of study performance; the respective most recent version of the respective OECD TG was applied

The classification for local toxicity and genotoxicity was performed in accordance with the *Globally Harmonized System of Classification and Labelling of Chemicals* (GHS; United Nations 2017) that has been implemented in EU *Regulation (EC) 1272/2008 on classification, labelling and packaging (CLP) of substances and mixtures* (EP and Council 2008)

*CLP* Classification, labelling and packaging, *GHS* Globally harmonised system for the classification and labelling of chemicals, *MP* Methyl paraben, *NOAEL* No-observed adverse effect level, *Ser*. Serious, *TG* Test guideline, *WoE* Weight-of-evidence

[a] All read-across data for butyl paraben (target substance) relate to *iso*-butyl paraben as source substance

[b] A further acute toxicity study was conducted as dermal exposure study in rabbits yielding NOAEL > 2000 mg/kg bw

[c] Study commissioned by Clariant Produkte (Deutschland) GmbH

[d] While methyl paraben exhibited in vitro clastogenicity with metabolic activation in an OECD TG 473 study, absence of in vivo clastogenicity in an OECD TG 478 study is overriding in the WoE evaluation so that the overall WoE conclusion is absence of genotoxicity

Neither the REACH Regulation (EP and Council 2006) nor the ensuing ECHA (2008,2013,2017b) guidance provides any specific parameters or thresholds to quantify structural similarity. To substantiate the hypothesis that the source substances and the target substance are structurally similar, the Tanimoto similarity coefficient (*T*_c_; Tanimoto 1958) was determined (Table 1) since it has proven suitable to establish structural similarity during grouping and read-across (Low et al. 2013; Hartung 2016; Mellor et al. 2019). The *T*_c_ can range from 0 (maximally dissimilar) to 1 (maximally similar) (Krasowski et al. 2009). Following previous recommendations (Xue et al. 1999; Keserü and Makara 2009; Oh 2012; Hartung 2016), *T*_c_ > 0.85 was assessed as indicating ‘high similarity’, and *T*_c_ > 0.7 as indicating ‘similarity’.

## Results

### Methyl paraben and propyl paraben: findings from higher-tier studies

All REACH information requirements to address human health concerns with respect to 90-day repeated-dose toxicity, developmental and reproductive toxicity (DART), and endocrine disrupting potential (EP and Council 2006) have been fulfilled for methyl paraben and propyl paraben. As described in further detail below, no adverse effects were recorded in any of the studies (Tables 6 and 7; Box 1).

**Table 6:**
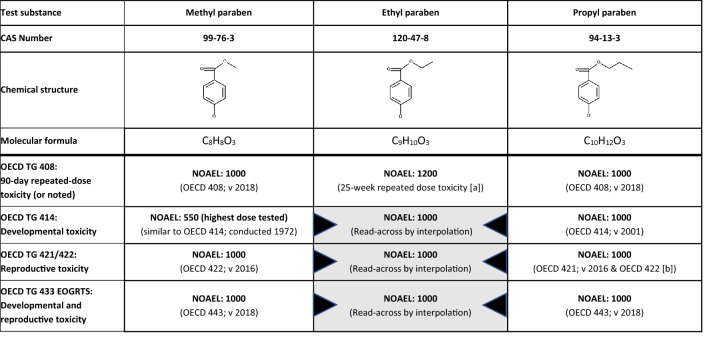
Overview of outcomes of repeated-dose toxicity studies and developmental and reproductive toxicity studies for methyl paraben and propyl paraben and application of read-across for ethyl paraben

The NOAEL is expressed in mg/kg body weight/day. This table contains data that are freely available on the ECHA dissemination portal (https://echa.europa.eu). Except for the historical studies (repeated-dose toxicity study for ethyl paraben, OECD TG 414-similar study for methyl paraben), all studies were commissioned by Clariant Produkte (Deutschland) GmbH in order to fulfil REACH information requirements. Unless noted otherwise, Wistar rats were used in all studies, and test material application was via oral gavage (see Table 4 for methodological details)

*EOGRTS* Extended one-generation reproductive toxicity study, *NOAEL* No-observed adverse effect level), *v* Version of OECD TG

[a] Feeding study using SD-JCL rats (Sado 1973; Liebert 1984)

[b] Feeding study using Wistar rats, conducted according to OECD TG 422 version of 1996 (Harlan 2012)

**Table 7:**
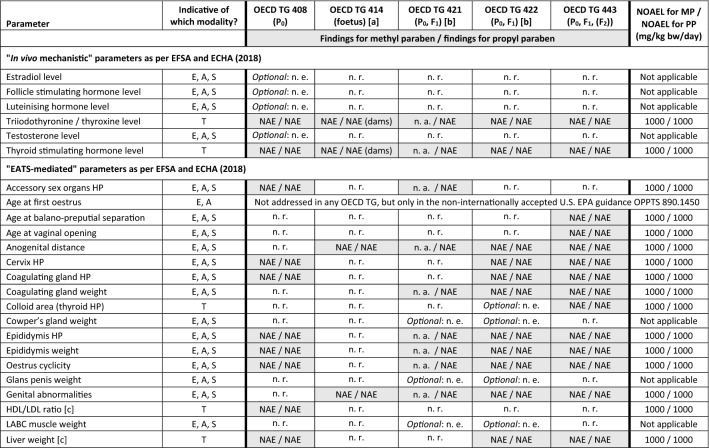

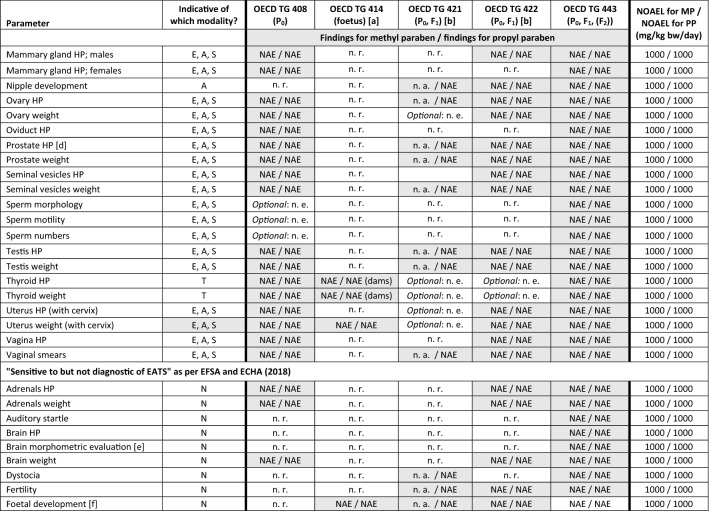

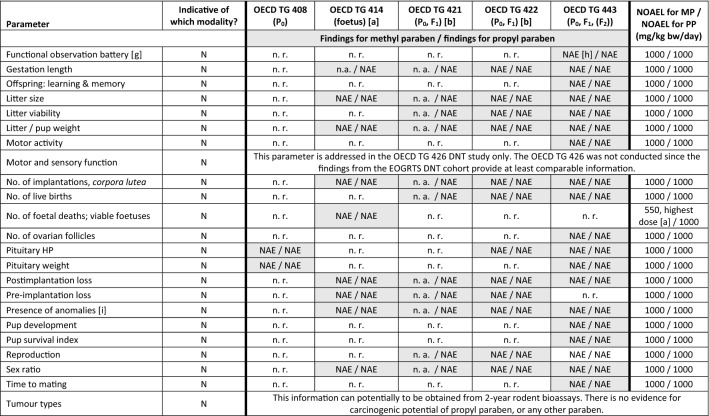
Higher-tier test results for methyl paraben and propyl paraben: parameters as per Table 14 in EFSA and ECHA (2018) that allow determining if a substance is, or is not, an endocrine disruptor

Colour legend: Grey shading: required as per Table 14 in EFSA and ECHA (2018); “optional”: as per that Table 14 and corresponding OECD TG

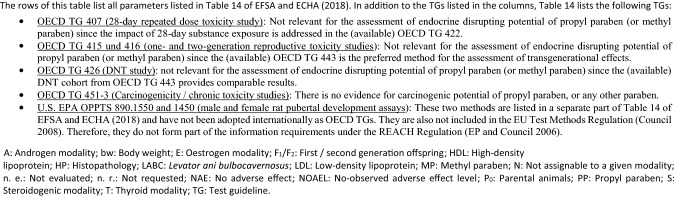

[a] For methyl paraben, a historical OECD TG 414-like study was available (Food and Drug Research Laboratories 1972) in which no findings were recorded up to the highest dose tested, i.e. 550 mg/kg bw/day. All relevant parameters included in OECD TG 414 were also addressed in OECD TG 408, 422 and 443 and consistently indicated a NOAEL of 1000 mg/kg bw/day. While “no. of foetal deaths; viable foetuses” is only addressed in OECD TG 414, this information is also obtained by “no. of implantations, *corpora lutea*” and “no. of live births” via OECD TG 422 and 443. Therefore, the NOAEL of 1000 mg/kg bw/day recorded for these parameters is overriding as compared to the NOAEL of 550 mg/kg bw/day recorded for “no. of foetal deaths; viable foetuses” for technical reasons

[b] For propyl paraben, OECD TG 421 was conducted with reduced animal numbers as dose range finding study for the subsequent OECD TG 443. For methyl paraben the (more comprehensive) OECD TG 422 also served as dose range finding study for the OECD TG 443. For propyl paraben, a historical OECD TG 422 was available (Harlan 2012) that had been conducted as feeding study (test groups: 0, 1500, 4500, 15,000 ppm). Harlan (2012) recorded a NOAEL of 15,000 ppm, corresponding to 981 and 1076 mg/kg bw/day, respectively, in the P_0_ males and females before pairing and 1125 mg/kg bw/day in the F_1_ generation

[c] “*These parameters are considered T-mediated, only when a change is observed in combination with other thyroid-related endpoints*” (EFSA and ECHA 2018)

[d] Including seminal vesicles and coagulating glands

[e] Also referring to quantitative morphometric brain assessments

[f] “*Or physical development of the foetuses?”* (EFSA and ECHA 2018)

[g] As described in Appendix A of OECD TG 443

[h] Evaluated by an integrated analysis of all neurodevelopmentally relevant data

[i] Including external, visceral, and/or skeletal abnormalities

**Table:** 

Box 1: Difference between Tables 6 and 7 presenting the findings from the higher-tier studies
Table 6 provides an overview of the outcomes of the higher-tier studies requested and conducted for methyl paraben and propyl paraben under the REACH Regulation (EP and Council 2006), by presenting the no-observed adverse effect level (NOAEL), which was determined based upon the overall outcome of each study. Further, this overview of findings is used in Table 6 to illustrate how the evidence is used to interpolate the hazard profile for ethyl paraben (see “Ethyl paraben: Interpolation from methyl paraben and propyl paraben”).
Table 7 provides a detailed list of the findings from these same studies. This table was structured in accordance with Table 14 of the European Food Safety Authority (EFSA) and ECHA *Guidance for the identification of endocrine disruptors in the context of Regulations (EU) No 528/2012 and (EC) No 1107/2009* (EFSA and ECHA 2018). Table 14 of this Guidance was selected as template for Table 7 since it provides an exhaustive data matrix for studies and parameters that allow determining if a substance is, or is not, an endocrine disruptor (see “No indication for endocrine disrupting potential of methyl paraben or propyl paraben (and thusly not for ethyl paraben either)”).

Sub-chronic (90-day) oral application of methyl paraben or propyl paraben to rats following OECD TG 408 did not induce any signs of systemic toxicity. Specifically, none of the rats showed any effects on reproductive organs, sperm parameters (males), oestrous cyclicity (females) or serum thyroid hormone levels. Hence, there were no indications for endocrine disrupting potential. For both substances, the no-observed adverse effect level (NOAEL) was set at 1000 mg/kg bw/day, i.e. the highest dose tested and limit dose as per OECD TG 408 (Tables 6 and 7).

In the prenatal developmental toxicity study (OECD TG 414), propyl paraben was orally administered to dams from the timepoint of implantation throughout pregnancy. As substance exposure occurs in a sensitive life stage, the OECD TG 414 covers parameters which are also predestined for the detection of endocrine disrupting properties. Endpoints include gestation, reduced gestation length, dystocia, implantation losses in dams, genital malformations, changes in anogenital distance in both sexes and/or increased nipple retention in males. Further, histopathological alterations of the reproductive organs and effects on the thyroid hormone system of the offspring are assessed. No treatment-related effects were observed in either the dams or pups treated with propyl paraben, and the NOAEL was set at 1000 mg/kg bw/day (Tables 6 and 7).

In the historical OECD TG 414-like study for methyl paraben (exposure from gestational day 6–15), no findings were recorded up to the highest dose tested, i.e. 550 mg/kg bw/day (Food and Drug Research Laboratories 1972). While this study did not extend across the entire gestational period and did not include testing up to 1000 mg/kg bw/day, the performance of a further OECD TG 414 following the most recent test protocol would have contradicted the 3Rs principle (EP and Council 2010): all endocrine disruption-relevant parameters included in OECD TG 414 are also addressed in OECD TG 408, 422 and 443 and consistently indicated absence of adversity up to the limit dose of 1000 mg/kg bw/day (Table 7). Of note, while the parameter ‘*no. of foetal deaths; viable foetuses*’ is only addressed in OECD TG 414, this same information is also obtained via OECD TG 422 and 443 by a combination of the parameters ‘*no. of implantations, corpora lutea*’ and ‘*no. of live births*’. Similarly, while the data from the historical OECD TG 414-like study did not inform on (reduced) gestation length, this information is also obtained via OECD TG 422 and OECD TG 443 (see also Table 7 with a juxtaposition of the parameters included in the different OECD TGs).

In the reproductive toxicity screening assays (OECD TG 421/422), no treatment-related effects were observed for either methyl paraben or propyl paraben in either the parental animals or the pups. The NOAEL for both substances was set at 1000 mg/kg bw/day. In the historical OECD TG 422 feeding study available for propyl paraben, a NOAEL of 15,000 ppm was recorded, which was the highest concentration tested and corresponded to 981 and 1076 mg/kg bw/day, respectively, in the parental males and females before pairing and to 1125 mg/kg bw/day in the first-generation offspring (Harlan 2012).

The extended one-generation reproductive toxicity study (OECD TG 443) is the most sensitive and most comprehensive study for detecting DART and/or endocrine disrupting effects that may occur as a result of pre‐ and postnatal substance exposure. This study provides information on gonadal function, the oestrus cycle, epididymal sperm maturation, mating, conception, gestation, parturition, lactation, weaning, and growth and development of the offspring. Further, the assessments included breeding up to the second-generation offspring and the two optional cohorts to investigate potential for DNT and DIT. Methyl paraben and propyl paraben elicited no toxicologically relevant alterations of any of the parameters addressed in the OECD TG 443 (Table 7; see Supplementary Information SI-1 for overview of findings that were assessed as non-toxicologically relevant). In this regard, they also did not exhibit DNT (evaluated by neurobehavioural testing, neurohistopathology, learning and memory testing) or DIT (evaluated by an integrated analysis of all immunologically relevant data including a T-dependent antibody response of a functional immune system (data not shown)). Since no adverse effects were observed for either methyl paraben or propyl paraben in the OECD TG 443 up to the limit dose, the NOAEL for both substances was set at 1000 mg/kg bw/day.

### Ethyl paraben: interpolation from methyl paraben and propyl paraben

The interpolation of missing data for ethyl paraben followed Scenario 5 of the ECHA (2017b) RAAF. Below, the assessment elements (AEs) for RAAF Scenario 5 are presented and discussed. Following the structure of the RAAF, it is distinguished between *Common AEs for category approaches* (see “Common assessment elements (AEs) for category approaches”) and Special AEs for Scenario 5 (see “Special assessment elements (AEs) for Scenario 5”). In the first part of the discussion (see “Appraisal of the read-across case to inform decision-making for ethyl paraben”), the read-across is completed by an overarching appraisal of the read-across case to inform decision-making.

#### Common assessment elements (AEs) for category approaches

##### AE C.1 Substance characterisation.

ECHA (2017b): “*This AE assesses whether the chemical identity and the impurity profile of each category member are sufficiently detailed for a scientific assessment of the category approach.*”

Comprehensive physical and chemical characterisation is available for both the source substances (methyl paraben, propyl paraben) and the target substance (ethyl paraben) as well as for the further category member butyl paraben (Table 3). All four parabens are mono-constituents, and they are all of very high purity, i.e. ≥ 98% for ethyl paraben (impurities: < 0.5% hydroxybenzoic acid and < 1.5% sum of unknown impurities), approx. 99.8% for methyl paraben and > 98% for propyl paraben. Both the target and the source substances exhibit very similar physical and chemical properties.

Assessment option; Score 5: Acceptable with high confidence.

##### AE C.2 Structural similarity and differences within the category.

ECHA (2017b): “*This AE confirms that all category members fulfil the criteria on required structural similarity and allowed structural differences detailed in the category definition.”*

The shorter-chained linear *n*-alkyl parabens methyl paraben (C_8_H_8_O_3_), ethyl paraben (C_9_H_10_O_3_), propyl paraben (C_10_H_12_O_3_) and butyl paraben (C_11_H_14_O_3_) are conjoined into a category on account of (1) their common functional group (all four are *n-*alkyl esters of *p*‐hydroxybenzoic acid, which are attached to a propyl group at the carboxylic acid functionality of the benzoic acid moiety); and (2) the incremental and constant change of their *n-*alkyl chain length. Without exception, all category members fulfil the criteria on required structural similarity and allowed structural differences detailed in the category definition.

The structural similarity of all four category members is supported by the Tanimoto index *T*_c_ (Table 3). For methyl paraben and propyl paraben, a *T*_c_ of 0.92 and 0.91, respectively, as compared to ethyl paraben was calculated indicating (very) high similarity and for butyl paraben a *T*_c_ of 0.82 as compared to ethyl paraben indicating similarity (close to the threshold of 0.85 indicating high similarity). The high *T*_c_ values reflect the increasing chain length between category members, while confirming that there are no inherent structural differences between the substances.

Assessment option; Score 5: Acceptable with high confidence.

##### AE C.3 Link of structural similarities and structural differences with the proposed regular pattern.

ECHA (2017b): “*This AE assesses whether a category hypothesis has been provided and whether it applies to all the category members.*”

All linear *n*-alkyl parabens have a common functional group and exhibit incremental and constant change of their *n*-alkyl chain length. The read-across hypothesis is based on the option of *“(bio)transformation to common compounds*” (i.e. *p*-hydroxybenzoic acid; see AE C.4). The source substances and the target substance (as well as the further category member butyl paraben) are considered to be biologically equivalent because they all follow the same metabolic pathways of enzymatic hydrolysis. Further, it is hypothesised that “*similar properties are observed for the different source substances; this may include absence of effects for every member of the category*” (ECHA 2017b).

Assessment option; Score 5: Acceptable with high confidence. The hypothesis applies in an unambiguous manner to all category members.

##### AE C.4 Consistency of effects in the data matrix.

ECHA (2017b): “*This AE further assesses whether the available data show that properties of the group members across the data matrix are consistent. Consideration is given to the nature and range of effects reported in the study(ies) to be read-across and in related properties identified in studies with the category members. This AE also checks whether effects differ in strength across the category members and whether this difference is characterised*.”

*Data matrix physico-chemical properties* (*Table* *3*): All four shorter-chained linear *n-*alkyl parabens show similar physico-chemical properties. Many of these properties show slight incremental trends that can be attributed to the continuously increasing alkyl chain length, but no fundamental change in any property (e.g. acid dissociation constant approx. 8.4; octanol–water partition coefficient ranging from approx. 2–3.5; all category members either only slightly soluble or insoluble in water).

*Data matrix acute toxicity, local toxicity, genotoxicity* (*Table* *5*): All four shorter-chained linear *n-*alkyl parabens were not toxic upon single administration (LD_50_ > 2000 mg/kg bw/day). They were not irritating to skin and eye (with the exception of the further category member butyl paraben that was assessed taking a conservative ‘worst-case’ approach based upon read-across to *iso*-butyl paraben). They were not sensitising to the skin and not mutagenic or clastogenic.

**Fig. 2:**
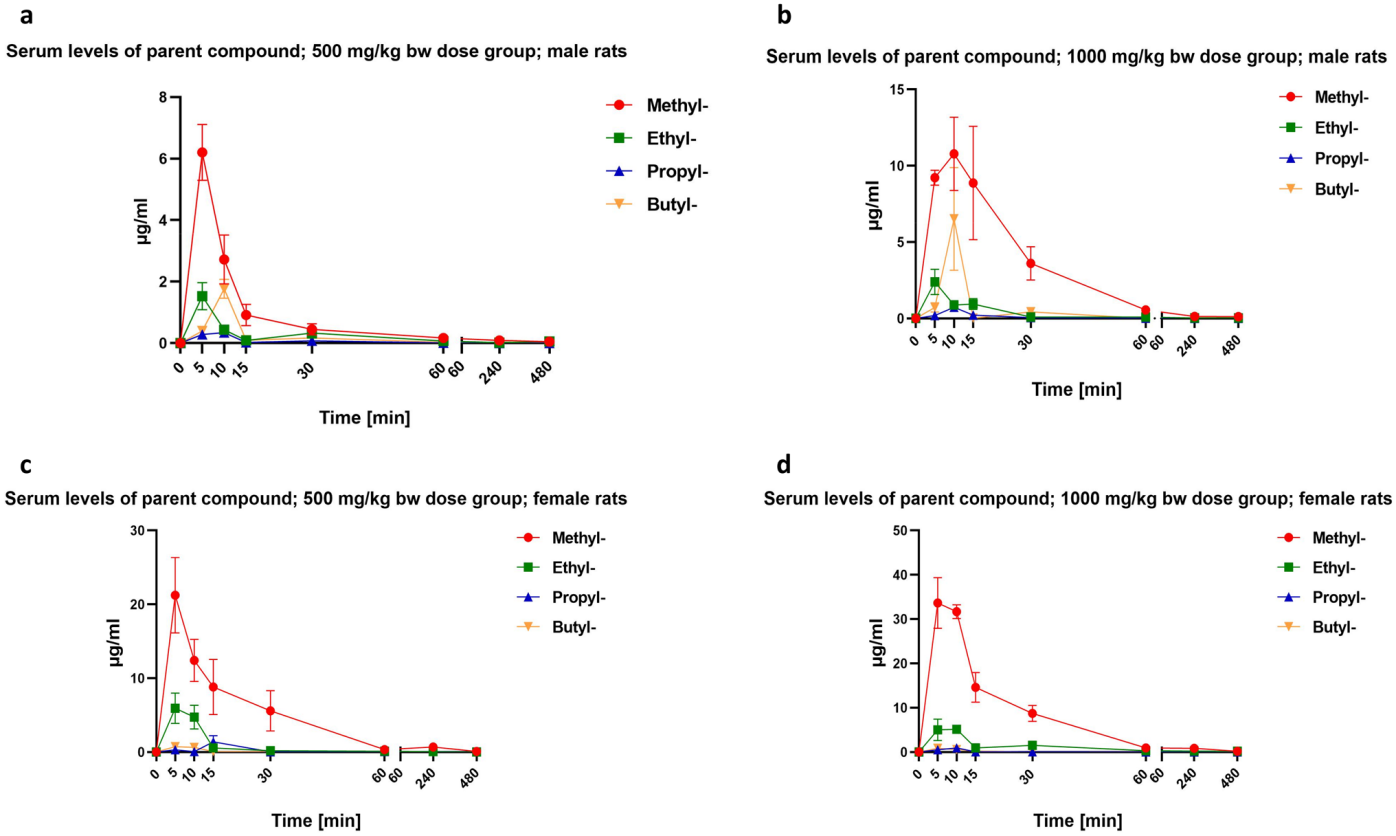
Outcome of in vivo toxicokinetics studies for methyl paraben, ethyl paraben, propyl paraben and butyl paraben (mean ± standard error of mean): elimination of parent compound from the blood stream of male and female rats **a** 500 mg/kg bw dose groups (10 males); **b** 1000 mg/kg bw dose groups (10 males); **c** 500 mg/kg bw dose groups (10 females); **d** 1000 mg/kg bw dose groups (10 females)

**Fig. 3:**
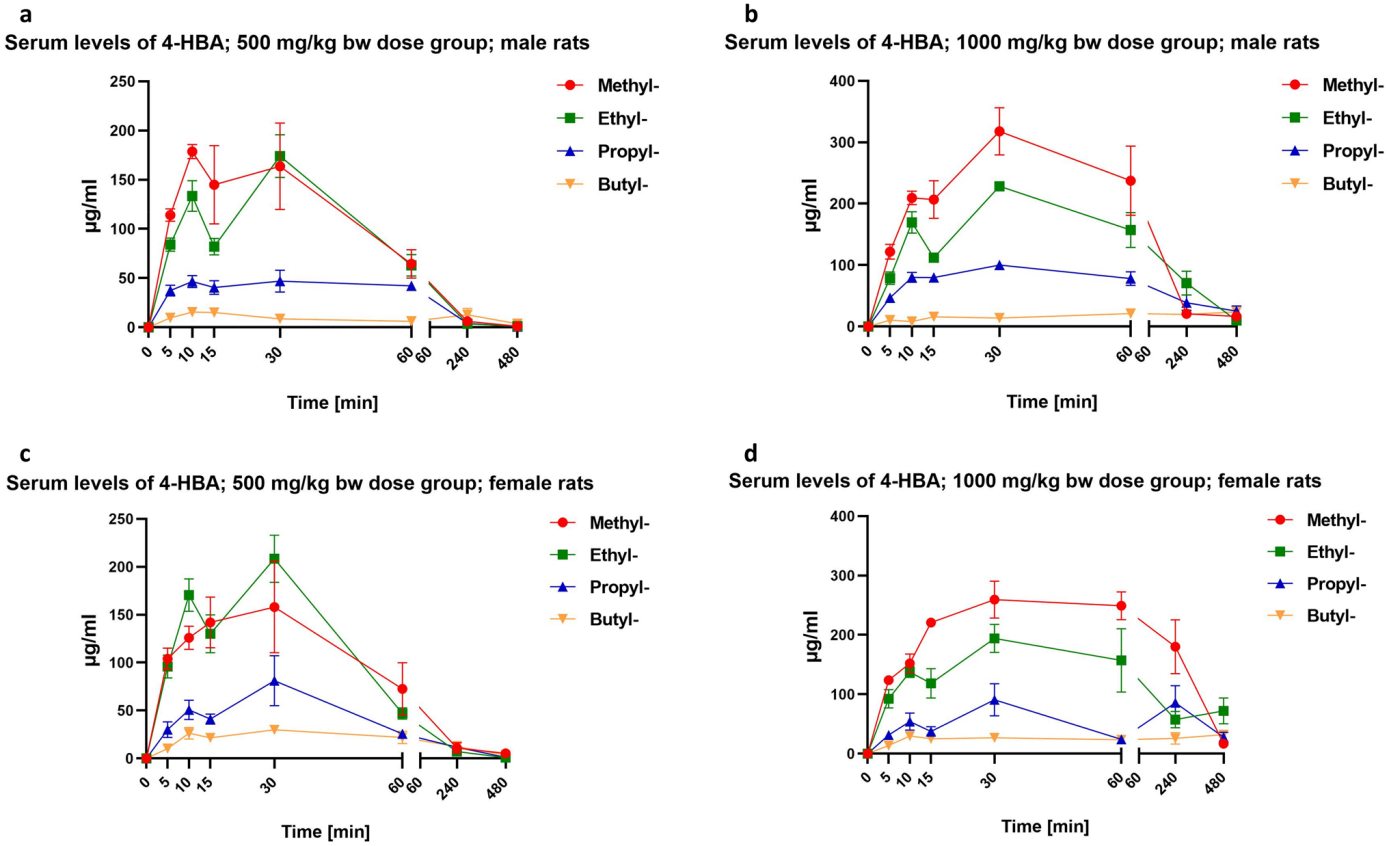
Outcome of in vivo toxicokinetics studies for methyl paraben, ethyl paraben, propyl paraben and butyl paraben (mean ± standard error of mean): elimination of the major metabolite 4 *p*-hydroxybenzoic acid (4-HBA) from the blood stream of male and female rats **a** 500 mg/kg bw dose groups (10 males); **b** 1000 mg/kg bw dose groups (10 males); **c** 500 mg/kg bw dose groups (10 females); **d** 1000 mg/kg bw dose groups (10 females)

*Data matrix in vivo toxicokinetic profile (Figs.* *2** and* *3*): Upon oral gavage application of either 500 or 1000 mg/kg bw test item, all four shorter-chained linear *n*-alkyl parabens showed a very similar toxicokinetic profile in the blood, as measured by ICP-MS (see “In vivo studies”):

1. The concentrations of the parent compounds increased rapidly in the blood within the first 5–10 min after oral gavage administration. A substantial portion of the overall exposure was seen within the first hour post-dosing; see parameter AUC_0–1 h_/AUC_0-t_ in Table 8. The AUC_0–1 h_/AUC_0–*t*_ reflects the area under the curve (AUC) during the first hour post-dosing relative to the AUC for the total period with quantifiable exposure. The highest AUC_0–1 h_/AUC_0–*t*_ values were recorded for the males of the methyl paraben high dose group (75%) and the females of the ethyl paraben low-dose group (70%). Further, methyl paraben consistently exhibited the highest maximum serum concentrations (*C*_max_) in both the male and female animals of both the low- and high-dose groups. In the methyl paraben high dose group, *C*_max_ of approx. 34 and 11 µg/mL were measured for the females and males, respectively. By comparison, the *C*_max_ in the ethyl paraben high-dose group was 5 and 2 µg/mL for the females and males, respectively, and the *C*_max_ in the males of the butyl paraben high-dose group was 7 µg/mL. The serum concentrations of propyl paraben (in both genders), just as butyl paraben in the female animals, were generally extremely low (Fig. 2a–d and Table 8). Taken together, methyl paraben exhibited the highest internal exposures (as compared to either ethyl paraben, propyl paraben or butyl paraben) in both the males and females of both the low- and high-dose groups.
2. All four shorter-chained linear *n*-alkyl parabens were eliminated from the blood stream very rapidly within the first hour of oral gavage administration in both the male and female rats of the 500 and 1000 mg/kg bw dose groups (Fig. 2a–d). Specifically, one hour after dosing, mean plasma concentrations of all four test items had decreased to less than 10% of the maximum concentration (Table 8; see parameter *C*_1h_/*C*_max_).
3. The elimination of the respective parent compounds from the bloodstream coincided with an increase in the serum concentration of the (common) major metabolite *p*-hydroxybenzoic acid in the male and female rats of both dose groups (Fig. 3a–d and Table 9). The *C*_max_ of *p*-hydroxybenzoic acid was always above 15 µg/mL and even reached 260 µg/mL and 318 µg/mL, respectively, in the females and males of the methyl paraben high-dose group. Also, the *C*_max_ was generally achieved after 30 min (Table 9).
  Notably, *p*-hydroxybenzoic acid (CAS No. 99-96-7) exhibits no systemic toxicity and no DART (https://echa.europa.eu/registration-dossier/-/registered-dossier/15944/2/1 [accessed 4 May 2020]). Further, during substance evaluation pursuant to Article 48 of the REACH Regulation, it was concluded that *p*-hydroxybenzoic acid does not exhibit any endocrine activity (ECHA 2014).
  Consistent with the findings for the parent compounds, the maximum concentrations of *p*-hydroxybenzoic acid were highest in the animals treated with methyl paraben, with subsequent ranking ethyl paraben > propyl paraben > butyl paraben. Just as butyl paraben serum concentrations were generally very low, also the concentrations of the metabolite *p*-hydroxybenzoic were very low in all butyl paraben dose groups.
4. *p*-Hydroxybenzoic acid was eliminated rapidly from the bloodstream within 4–8 h in both the male and female rats of the 500 and 1000 mg/kg bw dose groups (Fig. 3a–d and Table 9).

**Table 8:** Mean toxicokinetic parameters of methyl paraben, ethyl paraben, propyl paraben, and butyl paraben, following single oral administration to male and female Wistar rats

Test item	Dose (mg/kg)	Sex	*t*_max_ (h)	*C*_max_ (ng/mL)	SE_*C*_max_ (ng/mL)	*C*_1h_ (ng/mL)	*C*_1h_/C_max_ (%)	*t*_last_ (h)	*C*_last_ (ng/mL)	AUC_0-1 h_ (h ng/mL)	AUC_0–*t*_ (h ng/mL)	SE_AUC_0–*t*_ (h ng/mL)	AUC_0-1 h_/AUC_0–*t*_ (%)
Methyl paraben	500	f	0.08	21,233	5093	338	1.6	8.0	77.3	6450	9496	2520	68
		m	0.08	6199	911	172	2.8	8.0	48.6	1107	1792	222	62
	1000	f	0.08	33,630	5712	924	2.7	8.0	179	11,381	20,533	5036	55
		m	0.17	10,785	2404	552	5.1	8.0	122	4637	6153	1061	75
Ethyl paraben	500	f	0.08	5931	2054	116	2.0	8.0	23.9	1081	1540	283	70
		m	0.08	1539	436	71.6	4.7	8.0	70.6	320	632	88.8	51
	1000	f	0.17	5154	983	305	5.9	8.0	168	1659	3320	502	50
		m	0.08	2390	834	96.6	4.0	8.0	33.3	490	789	99.2	62
Propyl paraben	500	f	0.25	1387	831	68.0	4.9	8.0	136	291	1123	286	26
		m	0.17	345	116	23.0	6.7	4.0	13.6	94.9	150	30.2	63
	1000	f	0.17	940	374	20.2	2.1	8.0	11.6	156	317	58.1	49
		m	0.17	735	291	62.3	8.5	8.0	14.3	175	380	68.6	46
Butyl paraben	500	f	0.08	741	403	31.5	4.3	8.0	23.9	149	336	43.6	44
		m	0.17	1773	311	161	9.1	8.0	49.2	300	705	37.5	43
	1000	f	0.08	854	231	59.1	6.9	8.0	23.6	191	404	40.2	47
		m	0.17	6518	3364	373	5.7	8.0	18.4	860	1579	408	54

*AUC*_*0–1 h*_*/AUC*_*0–t*_ Area under the mean plasma concentration versus time curve from time zero up to one hour post-dosing/up to the last quantifiable concentration; both calculated by the trapezoidal rule, *C*_*1h*_ Mean plasma concentrations at one hour post-dosing, *C*_*last*_ Last quantifiable concentration or concentration at the last sampling time point, *C*_*max*_ Observed maximum plasma concentration, *f* Female, *m* Male, *SE_AUC*_*0–t*_ Standard error of the area under the mean concentration time curve, *SE_C*_*max*_ Standard error of data at *t*_max_, *t*_*last*_ Time of last analytically quantifiable plasma concentration or last sampling time point, *t*_*max*_ Time of occurrence of *C*_max_

**Table 9:** Mean toxicokinetic parameters of the major metabolite 4 *p*-hydroxybenzoic acid in male and female Wistar rats following single oral administration of methyl paraben, ethyl paraben, propyl paraben, and butyl paraben to male and female Wistar rats

Test item	Dose (mg/kg)	Sex	*t*_max_ (h)	*C*_max_(ng/mL)	SE_C_max_ (ng/mL)	*t*_last_ (h)	*C*_last_ (ng/mL)	AUC_0–*t*_(h ng/mL)	SE_AUC_0–*t*_ (h ng/mL) ara>
Methyl paraben	500	f	0.50	158,123	47,847	8.0	5364	278,371	62,684
		m	0.17	178,622	7170	8.0	1098	246,116	37,109
	1000	f	0.50	259,457	31,157	8.0	16,727	1,257,642	175,333
		m	0.50	317,839	38,414	8.0	16,619	702,894	110,645
Ethyl paraben	500	f	0.50	208,534	24,714	8.0	960	231,509	17,515
		m	0.50	173,968	21,818	8.0	1123	222,667	21,854
	1000	f	0.50	194,315	23,980	8.0	72,080	775,761	126,651
		m	0.50	228,645	4010	8.0	9689	667,635	87,442
Propyl paraben	500	f	0.50	81,117	26,161	8.0	1093	132,289	18,546
		m	0.50	46,972	11,062	8.0	803	120,713	8859
	1000	f	0.50	90,927	27,239	8.0	27,884	445,792	111,186
		m	0.50	100,040	5788	8.0	25,152	384,166	45,862
Butyl paraben	500	f	0.50	29,941	4848	8.0	3473	106,910	22,635
		m	0.17	15,585	3958	8.0	3652	70,937	23,605
	1000	f	8.00	32,146	3106	8.0	32,146	220,483	31,168
		m	8.00	23,925	3695	8.0	23,925	163,784	24,150

*AUC*_*0–t*_ Area under the mean plasma concentration versus time curve from time zero up to the last quantifiable concentration; calculated by the trapezoidal rule, *C*_*last*_ Last quantifiable concentration or concentration at the last sampling time point, *C*_*max*_ Observed maximum plasma concentration, *f* Female, *m* Male, *SE_AUC*_*0–t*_ Standard error of the area under the mean concentration time curve, *SE_C*_*max*_ Standard error of data at *t*_max_, *t*_*last*_ Time of last analytically quantifiable plasma concentration or last sampling time point, *t*_*max*_ Time of occurrence of *C*_max_

Interestingly, the highest concentration of the respective parent compounds was approx. threefold higher in the female rats than in the male rats, but nonetheless cleared almost completely within the first hour post-administration. By comparison, serum concentrations of *p*-hydroxybenzoic acid were in the same order of magnitude for both males and females (and even slightly higher in the high-dose males than in the high-dose females).

*Data matrix higher-tier studies (Tables* *6** and* *7*): In the comprehensive set of higher-tier OECD TG-conforming and GLP-compliant studies conducted for methyl paraben and propyl parabens under the REACH Regulation, no treatment-related effects were recorded for any of the parameters addressed. For all studies and endpoints, the NOAEL was set at the limit dose of 1000 mg/kg bw/day (see “Methyl paraben and propyl paraben: findings from higher-tier studies”).

Notably, these higher-tier studies that are included in REACH Annexes IX and X for substances manufactured or imported in quantities of 100 or 1000 tpy, or more, respectively, are not standard information requirements for butyl paraben that is manufactured or imported at 1–10 tpy (see “Evaluation of linear *n*-alkyl parabens under product-specific EU legislation and in the scientific literature” for a further discussion of published (mostly non-TG-conforming) studies addressing the potential DART of butyl paraben).

Assessment option; Score 5: Acceptable with high confidence. (Slight) trends in properties are observed only for the physico-chemical endpoints, whereas all endpoints related to local toxicity, systemic acute and repeated-dose toxicity and DART consistently show an absence of adverse effects.

##### AE C.5 Reliability and adequacy of the source study(ies).

ECHA (2017b): “*The AE investigates whether the test material(s) used correctly represent the source substance(s) in terms of purity and impurities and whether the study results are adequate for classification and labelling and/or risk assessment*.”

The test items methyl paraben and propyl paraben that were used in all studies correctly represent the source substance(s) in terms of purity and impurities (Table 3). All source studies (i.e. repeated-dose toxicity and DART studies) match the default REACH requirements and were conducted in accordance with the corresponding OECD TGs as well as in compliance with *Council Regulation (EC) No 440/2008 laying down test methods pursuant to the REACH Regulation* (Council 2008). Further, all source studies are fully GLP-compliant. Therefore, all study results that shall be used for read-across are both reliable and adequate for classification and labelling and risk assessment.

Assessment option; Score 5: Acceptable with high confidence.

##### AE C.6 Bias that influences the prediction.

ECHA (2017b): “*This AE assesses the extent to which it is clear from the documentation how other structurally similar substances have been considered as potential category members and generally whether other structurally similar substances could be used as additional category members. The AE addresses whether information available on these substances would result in a difference in the prediction of the properties under consideration for the target substance. This AE also addresses whether the source study(ies) used as the basis for the prediction correspond(s) to the reliable study(ies) giving rise to the highest concern for the properties under consideration.”*

As explained in “Grouping and read-across as practicable approach to minimise animal testing”, focus of the present article is on the shorter-chained linear *n*-alkyl parabens and an interpolation of missing data for ethyl paraben (as target substance) using measured values from other members on both sides of that member within the defined category spectrum (i.e. methyl paraben and propyl paraben as source substances) as requested in ECHA (2008) and OECD (2014). [The extrapolation of missing data for butyl paraben is briefly addressed in the discussion (see “Tentative extrapolation of missing data for butyl paraben”).]

Data for parabens with longer *n*-alkyl moieties (pentyl paraben, hexyl paraben, heptyl paraben, etc.) are not available on the ECHA dissemination portal (https://echa.europa.eu/). However, the database for repeated-dose toxicity and DART is both complete and consistent for methyl paraben and propyl paraben, i.e. the two category members on both sides of the target substance ethyl paraben. Consistently, the data for methyl paraben and propyl paraben indicate the identical toxicokinetic profile, the identical metabolic pathway and absence of toxicity up to the limit dose. Similarly, the identical toxicokinetic profile and identical metabolic pathway are indicated for the further category member butyl paraben that has one CH_2_-unit more than propyl paraben (see also Subsection AE 5.5 in “Special assessment elements (AEs) for Scenario 5”, that no non-common metabolites are formed during the metabolism of linear *n*-alkyl parabens). Therefore, any deliberations to conduct higher-tier studies for longer-chained linear *n*-alkyl parabens—for the sole purpose to substantiate read-across for ethyl paraben (since these longer-chained parabens are not registered under REACH) - would contradict the 3Rs principle implemented in Directive 2010/63/EU (EP and Council 2010) or the provisions of Article 25(1) of the REACH Regulation (EP and Council 2006) that requires that testing on vertebrate animals shall be undertaken only as a last resort.

Assessment option; Score 5: Acceptable with high confidence.

#### Special assessment elements (AEs) for Scenario 5

##### AE 5.1 Formation of common (identical) compound(s).

ECHA (2017b): “*This AE covers only the formation of the common compound(s) as it is addressed in the hypothesis, irrespective of their effects. Convincing evidence has to be provided that the common compound(s) are formed from the category members. If the scientific explanation for the formation of the common compound(s) is missing for one or more category members, it has to be assessed whether this has any impact on the prediction of the properties under consideration*.”

All four *n*-alkyl parabens showed the identical metabolic pathway and a very similar toxicokinetic profile: they were very rapidly eliminated from the blood stream within the first hour of administration (both in the 500 and 1000 mg/kg bw dose groups). Concordantly, the concentration of the non-toxic major metabolite *p*-hydroxybenzoic acid rose that was then eliminated within 4–8 h (see “Common assessment elements (AEs) for category approaches”; AE C.4).

Assessment option; Score 5: Acceptable with high confidence.

##### AE 5.2 The biological target(s) for the common compound(s).

ECHA (2017b): “*This AE investigates how the (bio)transformation of source and target substances to the common compound(s) results in the exposure of the same biological target(s) and whether the same type of effects are induced in the same biological targets by the common compound(s) throughout the category*.”

The very rapid elimination of all four shorter-chained linear *n*-alkyl parabens from the bloodstream, that is almost complete within one hour after oral gavage administration, consistently limits or even prevents relevant in vivo exposure to any biological target. The almost identical kinetic behaviour thus entails a comparable systemic exposure profile of all parabens tested. Similarly, the simultaneously formed common major metabolite *p*-hydroxybenzoic acid is also eliminated rapidly within 4–8 h so that there is no relevant exposure to any biological target. Finally, *p*-hydroxybenzoic acid is non-toxic and does not exhibit endocrine activity. Therefore, it does not elicit any effects on biological targets.

Assessment option; Score 5: Acceptable with high confidence.

##### AE 5.3 Exposure of the biological target(s) to the common compound(s).

ECHA (2017b): “*This AE focuses on whether the similarity in the exposure of the biological targets to the common compound(s) is established*.”

Based on the almost identical kinetic behaviour, none of the four shorter-chained linear *n*-alkyl parabens, nor their common and non-toxic major metabolite *p*-hydroxybenzoic acid, exhibits any toxicologically relevant exposure to any biological target.

Assessment option; Score 5: Acceptable with high confidence.

##### AE 5.4 The impact of parent compounds.

ECHA (2017b): “*The (bio)transformation of the target and source substances may not be immediate and/or complete… This AE investigates whether the systemic availability of the parent compounds and of their impurities have been addressed and its impact on the prediction of the property under consideration has been assessed. For local biological targets, the exposure to the parent compounds at the site of contact has to be considered*.”

The (bio)transformation of the target and source substances is immediate and complete. Both the target and source substances are of very high purity. The low fraction of impurities mainly constitutes the common and non-toxic major metabolite *p*-hydroxybenzoic acid, which is also rapidly eliminated from the bloodstream within 4–8 h after oral gavage administration.

Assessment option; Score 5: Acceptable with high confidence.

##### AE 5.5 Formation and impact of non-common compounds.

ECHA (2017b): “*The formation of common compound(s) often goes together with the formation of non-common compound(s) and possible intermediates, which form the common compound(s). Source and/or target substances can also be (bio)transformed through other pathways than that leading to the formation of the common product(s), and which generate additional non-common compounds. This AE examines whether the formation of non-common compounds (including possible intermediates) formed through such other pathways and their possible impact on the prediction of the property under consideration have been considered*.”

All parabens are readily metabolised back to *p*-hydroxybenzoic acid by esterases in different tissues. No non-common metabolites are formed during the metabolism of linear *n*-alkyl parabens; an additional metabolic pathway, that is also common to all parabens, is their glucuronidation and/or sulfation, leading to elimination of the respective conjugates in the urine (Abbas et al. 2010; Aubert et al. 2012; Zhao et al. 2014; Moos et al. 2016).

Assessment option; Score 5: Acceptable with high confidence.

## Discussion

### Appraisal of the read-across case to inform decision-making for ethyl paraben

#### The very high structural similarity of the source substances and the target substance is confirmed

The source substances methyl paraben and propyl paraben exhibit very high structural similarities of *T*_c_ of 0.92 and 0.91 for, respectively, as compared to the target substance ethyl paraben. These very high *T*_c_ values reflect the increasing chain length between category members and confirm absence of inherent structural differences. Further, the physical and chemical characterisation of both the source substances and the target substance (and of butyl paraben) is complete and indicates very similar physical and chemical properties for all shorter-chained linear *n*-alkyl parabens with slight incremental trends that can be attributed to the continuously increasing alkyl chain length.

#### The read-across hypothesis *“(bio)transformation to common compounds*” is confirmed

The in vivo toxicokinetic screening studies consistently showed that all four shorter-chained linear *n*-alkyl parabens were taken up systemically very rapidly after oral gavage administration to rats and that they were eliminated very rapidly from the bloodstream within one hour after administration. Also, for all four test items, the major metabolite *p*-hydroxybenzoic acid was cleared rapidly from the blood stream within 4–8 h. *P*-hydroxybenzoic acid does not exhibit systemic toxicity or DART, and it is also not endocrine active (see “Common assessment elements (AEs) for category approaches”; AE C.4). The consistently higher uptake of all four shorter-chained linear *n*-alkyl parabens by the female rats, as compared to the male rats, was also observed by Aubert et al. (2012) in a toxicokinetics study using radiolabelled parabens. Aubert et al. (2012) did not provide a biological explanation for this gender difference.

Notably, the enzymes involved in the metabolism of parabens, i.e. carboxylesterases, glucuronidases and sulfotransferases, are ubiquitous and highly conserved across species, while exhibiting some quantitative differences, e.g. with respect to the activity of specific isoenzymes (Mizukawa et al. 2017; Moos et al. 2016; Wang et al. 2018). Irrespective of the species studied, the metabolism of parabens results in hydrolysis to the principal metabolite *p*-hydroxybenzoic acid, which may then be conjugated for subsequent urinary excretion (EMA 2015). These observations support cross-species extrapolations.

#### The hypothesis *“no relevant differences in predicted properties are observed for several source substances*” is confirmed

The rapid elimination of all shorter-chained linear *n*-alkyl parabens and their major metabolite *p*-hydroxybenzoic acid explains the absence of systemic toxicity, DART, and endocrine disrupting potential upon administration to male and female Wistar rats.

All REACH information requirements of relevance to assess potential for systemic toxicity, DART and endocrine disruption in the context of human health hazard assessment have been fulfilled for methyl paraben and propyl paraben. The higher-tier (i.e. REACH Annex IX–X) studies were conducted following internationally agreed OECD TGs and in full compliance with the principles of GLP. Thereby, the relevance, reliability and repeatability of study outcomes is ensured. Consistently, no adverse effects were recorded for methyl paraben and propyl paraben up to the limit dose of 1000 mg/kg bw/day (Table 7). Hence, all studies consistently indicate that methyl paraben and propyl paraben are (1) devoid of repeated-dose systemic toxicity; (2) devoid of DART; and (3) devoid of endocrine disrupting properties.

For both methyl paraben and propyl paraben, the NOAEL was set at 1000 mg/kg bw/day with regard to systemic repeated-dose toxicity and DART. Similarly, there is no indication for endocrine disrupting potential of methyl paraben or propyl paraben in any of the parameters addressed (Table 7); see further discussion in “No indication for endocrine disrupting potential of methyl paraben or propyl paraben (and thusly not for ethyl paraben either)”.

#### For ethyl paraben, a NOAEL of 1000 mg/kg bw/day for systemic repeated-dose toxicity and DART is interpolated

Taken together, all available data consistently confirm that read-across of systemic repeated-dose toxicity, DART from methyl paraben and propyl paraben (as source substances) to ethyl paraben (as target substance) is acceptable with high confidence.

#### Read-across of the findings for methyl paraben, ethyl paraben and propyl paraben to the respective sodium salts also appears justifiable

Similarly, read-across of the findings for methyl paraben, ethyl paraben and propyl paraben to the respective sodium salts, i.e. Na-methyl paraben, Na-ethyl paraben and Na-propyl paraben, appears justifiable on account of their very high *T*_c_ of 0.98 as compared to the respective paraben (Supplementary Information Table SI-2). Also, the sodium salts of the parabens exhibit very similar physico-chemical properties (Table SI-2), and, just as their parent compounds, no acute toxicity, local toxicity or genotoxicity potential (Supplementary Information Table SI-3). Methyl paraben, ethyl paraben and propyl paraben are each grouped with their respective sodium salts following the analogue approach, which is employed for the grouping of few, very structurally similar substances (OECD 2014; ECHA 2017b). Thus, a NOAEL of 1000 mg/kg bw/day for systemic repeated-dose toxicity and DART, as well as absence of endocrine disrupting potential, is predicted for Na-methyl paraben, Na-ethyl paraben and Na-propyl paraben.

### No indication for endocrine disrupting potential of methyl paraben or propyl paraben (and thusly not for ethyl paraben either)

Neither the REACH Regulation (EP and Council 2006) nor any of the ensuing ECHA documents provide any guidance for how to identify endocrine disrupting properties. By contrast, for biocidal products and plant protection products, criteria for the determination of endocrine disrupting properties have been implemented in the Commission (2017) and Commission (2018) Regulations, respectively (see Supplementary Information SI-4 for details). These criteria widely follow the WHO IPCS (2002) definition for an endocrine disruptor. Further, the EFSA and ECHA (2018) Endocrine Disruptor Guidance provides detailed provisions for how to determine if a substance meets, or does not meet, the Commission (2017, 2018) endocrine disruptor criteria. Even though this guidance was developed for biocidal products and plant protection products, it explicitly includes the fulfilment of information requirements under REACH [see page 57 of EFSA and ECHA (2018)].

According to the endocrine disruptor criteria (Commission 2017, 2018) and the WHO IPCS (2002) definition, endocrine disrupting properties can only be determined if *adverse* effects are observed in living organisms (in vivo). In this regard, it is noteworthy that while *n*-alkyl parabens have been observed to activate oestrogen receptors in vitro (see “Follow-up of concern for endocrine disrupting potential of parabens”), all recorded effects remained many orders of magnitude lower than those of the (non-toxic) natural oestrogen 17ß-estradiol. Also, the OECD CF Level 2 in vitro hormone receptor assays are insufficient to determine if a substance has endocrine disrupting properties, because they neither inform on the development of adverse effects nor allow investigating biotransformation as pivotal aspect of paraben behaviour in living organisms. By comparison, the OECD CF Level 4 and 5 studies presented herein (OECD TG 408, 414, 421/422 and 443) do not provide any indication that any endocrine activity (if it were present in vivo) would be sufficiently pronounced to overwhelm the physiological adaptive capacities of endocrine systems leading to adversity as indispensable prerequisite for endocrine disruption.

Also, the pattern of effects seen for methyl paraben and propyl paraben (oestrogen receptor activation in vitro, but no adverse effects in vivo) is not consistent with the pattern of effects of known oestrogen agonists, which includes ovarian malfunction evidenced by, e.g. reduced numbers of *corpora lutea* and large antral follicles (Biegel et al. 1998; NTP 2010). Similarly, physiologically active oestrogens would be expected to affect male secondary sex organs (Yamasaki et al. 2003, 2004). By contrast, the higher-tier studies presented herein, and a recent two-generation reproductive assessment rat feeding study assessing butyl paraben (Hubbard et al. 2020); see below), did not provide any indication for effects of parabens on male secondary sex organs.

The view that in vitro oestrogen receptor activation potencies of the linear *n*-alkyl parabens are not sufficiently high to act via an oestrogenic mode-of-action in humans (Watanabe et al. 2013; Borgert et al. 2018) is further supported by aggregate exposure assessments in humans: for methyl paraben, ethyl paraben and propyl paraben, aggregate exposure assessments addressing oral, dermal and inhalation exposures to personal care products, medicinal products and food (as three major sources of exposure) revealed estimates of approx. 7.2 and 4.5 mg/kg bw/day for human external and internal exposures, respectively (Brand et al. 2017). Compared to the NOAELs of 1000 mg/kg bw/day indicating absence of endocrine disruption-related effects that were established in the repeated dose toxicity and DART studies, the aggregated external exposure of humans to parabens is still approx. 140 times lower. Further, parabens do not bioaccumulate since they are eliminated from the body very rapidly (Soni et al. 2005).

Section 3.4.1 of EFSA and ECHA (2018) describes a ‘sufficient dataset’ to support a conclusion on the *absence* of adversity mediated by (o)estrogen, androgen, thyroid, and/or steroidogenic (acronym: EATS) pathways—and hence on the absence of endocrine disrupting properties (Box 2). Further, Table 14 in EFSA and ECHA (2018) that was used as template for Table 7 in the present article, provides an exhaustive data matrix for studies and parameters that allow determining if a substance is, or is not, an endocrine disruptor. As shown in Box 2, the dataset described in Sect. 3.4.1 of EFSA and ECHA (2018) is indeed ‘sufficient’ for methyl paraben and propyl paraben, and all parameters listed in Table 7 consistently show that methyl paraben and propyl paraben are not endocrine disruptors. For this reason, absence of endocrine disrupting potential is also interpolated for ethyl paraben.

**Table:** 

Box 2: Sufficient dataset to support a conclusion on the *absence* of EATS-mediated adversity as per Sect. 3.4.1 in EFSA and ECHA (2018)
“*…to have the EAS-mediated adversity with regard to humans and mammals (as non-target organisms) sufficiently investigated, all the data requirements of the specific Regulations, must be fulfilled. This should include all the ‘EAS-mediated’ parameters foreseen to be investigated in an extended one-generation reproductive toxicity study; OECD TG 443; with cohort 1a/1b including the mating of cohort 1b to produce the F2 generation*…”.
The dataset to support absence of EAS-mediated adversity is sufficient for methyl paraben and propyl paraben.
To have thyroid-mediated adversity sufficiently investigated, “*the thyroid parameters foreseen to be investigated in OECD TG 407, 408, 409 (or the one-year dog study, if available), 416 (or 443 if available) and 451–3 should have been measured…”.*
This list includes:
*OECD TG 408 and OECD TG 443*, which are both available for methyl paraben and propyl paraben.
*OECD TG 407 (28-day repeated dose toxicity study)*, which is covered by the available OECD TG 422 (reproductive toxicity screening study combined with 28-day toxicity).
*Non-rodent studies* (i.e. the OECD TG 409 90-day non-rodent oral toxicity study or the one-year dog study). In this regard, the REACH Regulation indicates that the decision to perform a study on a second, non-rodent species should be based on the outcome of the first species and all other relevant available data. The comprehensive data set available for both methyl paraben and propyl paraben consistently indicates absence of systemic toxicity and DART. Therefore, further testing in a second species would contradict the animal welfare principles laid down in Article 25(1) of the REACH Regulation (see also “Background”).
*OECD TG 451-3 carcinogenicity studies* As per REACH Annex X, a carcinogenicity study may be requested for a substance with widespread dispersive use if it is classified as germ cell mutagen category 2 or if there is evidence from the repeated-dose study(ies) that the substance can induce hyperplasia and/or pre-neoplastic lesion. None of these criteria apply for methyl paraben, ethyl paraben, or propyl paraben.
The dataset to support absence of thyroid-mediated adversity is sufficient for methyl paraben and propyl paraben.

### Tentative extrapolation of missing data for butyl paraben

As clearly denoted in ECHA (2008), interpolation is preferred over extrapolation, and the extrapolation of missing data for butyl paraben (with a linear *n*-alkyl chain with one CH_2_-unit more than propyl paraben) is not the focus of the present article. Nonetheless, it is noteworthy that the findings from the in vivo toxicokinetics screening assays consistently indicate that all four shorter-chained linear *n*-alkyl parabens, as well as their common and non-toxic major metabolite *p*-hydroxybenzoic acid, are cleared from the bloodstream very rapidly. Indeed, the butyl paraben test groups exhibited the lowest maximum serum concentrations as compared to those recorded for the three other parabens. Also, the maximum concentrations of the common and non-toxic major metabolite *p*-hydroxybenzoic acid were lowest in the butyl paraben test groups. Therefore, it is most likely that also butyl paraben is not systemically bioavailable for a sufficiently long period of time to elicit adverse effects on systemic target organs.

Taken together, the findings presented in this article indicate that extrapolation of the missing data on systemic repeated-dose toxicity and DART for butyl paraben from the corresponding data available for methyl paraben and propyl paraben is likely justifiable.

### Evaluation of linear n-alkyl parabens under product-specific EU legislation and in the scientific literature

While the manufacture and safe occupational handling of parabens is regulated under the REACH Regulation (EP and Council 2006) in the EU, the safety of consumers that use products containing parabens (e.g. food, pharmaceuticals and cosmetics) is regulated by product-specific regulations (Box 3).

**Table:** 

Box 3: Product-specific EU regulations of relevance for the safety assessment of products containing parabens
For food: *Regulation (EC) No 178/2002 laying down the general principles and requirements of food law, establishing the EFSA and laying down procedures in matters of food safety* (EP and Council 2002) with EFSA as main agency.
For pharmaceuticals: *Regulation (EC) No 726/2004 laying down Community procedures for the authorisation and supervision of medicinal products for human and veterinary use and establishing a European Medicines Agency* (EMA; EP and Council 2004) with EMA as scientific agency.
For cosmetics: *Regulation (EC) No 1223/2009 on cosmetic products* (EP and Council 2009) with the Scientific Committee on Consumer Safety (SCCS) as independent body to provide advice on the safety of non-food consumer products.

EFSA, the European Medicines Agency (EMA), and the Scientific Committee on Consumer Safety (SCCS) have published opinions on the safety of parabens in food, pharmaceuticals and cosmetics, respectively:

- EFSA (2004): *Opinion of the Scientific Panel on Food Additives, Flavourings, Processing Aids and Materials in Contact with Food on p-hydroxybenzoates*;
- SCCS (2013): *Updated scientific opinion on propyl- and butylparaben*;
- EMA (2015): *Reflection paper on the use of methyl- and propyl-paraben as excipients in human medicinal products for oral use*.

The REACH Regulation emphasises that the work by other agencies should be considered when evaluating substances under REACH [e.g. “*This Regulation should otherwise be without prejudice to the competence conferred on the EMA, the EFSA and the Advisory Committee on Safety, Hygiene and Health Protection at Work by Community legislation*”; Preamble No. 70 in EP and Council (2006)]. Accordingly, the three opinions by EFSA (2004), SCCS (2013) and EMA (2015) are to be considered when assessing the respective parabens under the REACH Regulation. Generally, all three opinions confirm that the use of methyl paraben, ethyl paraben, propyl paraben and butyl paraben in the respective products is safe to the consumer provided that, e.g., specific concentrations are not exceeded.

For *methyl paraben*, EFSA (2004) and EMA (2015) conclude that it has not been associated with adverse effects on the male and female reproductive organs in juvenile rats or in developmental toxicity studies, and a NOAEL of 1000 mg/kg bw/day is set as point of departure for the safety evaluation. Similarly, SCCS (2013) reiterated its earlier conclusion that the continued use of methyl paraben as preservative in cosmetics at the maximum authorised concentration was considered safe for human health.

For *ethyl paraben*, a NOAEL of 1000 mg/kg bw/day is determined in EFSA (2004), and its safety is reconfirmed in SCCS (2013), whereas this paraben is not included in EMA (2004).

For *propyl paraben*, SCCS (2013) and EMA (2015) generally set a NOAEL of 1000 mg/kg bw/day based upon key studies by Gazin et al. (2013) and Pouliot (2013). Findings from the unpublished study report by Pouliot (2013) have since been published by Sivaraman et al. (2018). Further, EMA (2015) determined a “conservative” no-observed effect level (NOEL) of 100 mg/kg/day for propyl paraben “based on the results on the female reproductive system,” (EMA 2015). As compared to a NOAEL, which indicates absence of adversity, a NOEL indicates absence of any effect. Therefore, a NOEL is generally lower than a NOAEL. The findings referred to in EMA (2015) to support the NOEL of 100 mg/kg/day, and similarly in SCCS (2013), relate to earlier onset of puberty and increased uterus weight, albeit without concomitant effect on the histology of reproductive tissues, oestrous cyclicity, mating and fertility, and maternal performance—for which reason a conservative NOEL, but not a NOAEL, was established.

For *butyl paraben*, not considered in EMA (2015), SCCS (2013) concluded that its use as preservative in finished cosmetic products is safe to the consumer, as long as specific concentrations are not exceeded. However, in February 2020, the Danish Environmental Protection Agency (DK EPA 2020) submitted a proposal for identifying butyl paraben as Substance of Very High Concern pursuant to REACH Article 57. The main adverse effects purported by the DK EPA (2020) are irreproducible reduced sperm count and sperm quality observed in rodent studies addressing perinatal substance exposure. The DK EPA (2020) cites non-TG-conform studies by Kang et al. (2002), Zhang et al. (2014), Boberg et al. (2016), and Guerra et al. (2017) to support these assumptions. It is important to note that the cited studies are not conclusive (see Supplement SI-5 for further discussion) since the findings were not all reproducible (especially with respect to sperm counts and sperm quality), and since they provided no clear indication for endocrine disrupting potential of butyl paraben. Further, the cited studies generally only included substance exposure during gestation and lactation.

By contrast, recent evidence from a two-generation reproductive assessment rat feeding study conducted within the U.S. National Toxicology Program (Hubbard et al. 2020) showed no association between oral exposure to 5,000 ppm, 15,000 ppm and 40,000 ppm butyl paraben and adverse alterations of fertility, fecundity, pubertal attainment or any reproductive parameters in the parent, first, or second generation (but exposure-dependent increases in liver weight and incidences of non-neoplastic liver lesions). Notably, in this study, already the mid-dose (15,000 ppm) generally exceeded the OECD limit dose of 1000 mg/kg bw/day by up to twofold, whereas the high-dose group (40,000 ppm) exceeded it by at least 2.5-fold and up to 6.7-fold (depending on the animals’ life cycle stage, e.g., gestation, lactation). For example, during lactation, average uptake of the first-generation females of the high-dose group was 6,709.4 mg/kg bw/day. For a 60-kg breastfeeding mother, this would correspond to an intake of more than 400 g pure butyl paraben every single day over the course of the breastfeeding period (and approx. 200 g butyl paraben every single day during all other periods of her life). According to the authors, these indications of hepatic toxicity may be associated with a sustained adaptive response in the first-pass organ as a result of long-term exposure to unrealistic extreme dosages and thus do not provide a real biological concern. Hence, the findings from this recent two-generation study, showing that “*all assessed in vivo measures of potential estrogenic and anti-androgenic activity were unperturbed at exposure levels that far exceed those currently used for butyl paraben risk assessments and margin of safety determinations*” (Hubbard et al. 2020), support the conclusion drawn in the present article, that butyl paraben does not exhibit any endocrine disrupting properties. Also, the findings from the standardised two-generation study, that have now been published by Hubbard et al. (2020), provide a strong foundation to rebut the concerns expressed in DK EPA (2020) that are based upon inconclusive, non-TG-conform studies.

## Conclusion

As discussed by Barlow et al. (2015), it is an essential aspect of the EU REACH Regulation to use the collated data for hazard-based substance classification and labelling following the provisions of *Regulation (EC) No 1272/2008 on Classification, Labelling and Packaging of substances and mixtures* (EP and Council 2008). For substances with endocrine disrupting properties, EU legislation specifically mandates regulation via a hazard-based approach (Brescia 2020). In line with these provisions, the present article has focussed on hazard identification of methyl paraben, ethyl paraben and propyl paraben (while also considering the further category member butyl paraben and briefly referring to the sodium salts of methyl paraben, ethyl paraben and propyl paraben).

For methyl paraben and propyl paraben, all higher-tier studies of relevance for the determination of repeated-dose toxicity, DART and endocrine disrupting potential have been requested under REACH, and the findings from these studies have been presented herein. For both methyl paraben and propyl paraben, the NOAEL with regard to repeated-dose toxicity and DART was set at 1000 mg/kg bw/day.

For ethyl paraben, a NOAEL of 1000 mg/kg bw/day for repeated-dose toxicity and DART was estimated by interpolation from methyl paraben and propyl paraben, i.e. the two category members on both sides of the target substance (ECHA 2008; OECD 2014). The chemical category of shorter-chained linear *n*-alkyl parabens is founded on their high structural similarity (the only difference between these parabens is their increasing chain length) and, importantly, on their common metabolic pathway. All category members exhibit similar physico-chemical properties and similar acute toxicity, local toxicity and genotoxicity potential. The rat toxicokinetics screening studies consistently showed that due to the very rapid elimination of these parabens and their major metabolite (that is further non-toxic), systemic target organs and tissues are not exposed to these compounds for a sufficiently long period of time for effects to evolve. Due to the consistency of the findings, the interpolation of a NOAEL of 1000 mg/kg bw/day for repeated-dose toxicity and DART for ethyl paraben is assessed as acceptable with high confidence. Performing the corresponding higher-tier studies, that encompass large numbers of animals (Table 4), for the hazard assessment of ethyl paraben would breach the 3Rs principle implemented in Directive 2010/63/EU (EP and Council 2010) and Article 25(1) of the REACH Regulation (EP and Council 2006) that requires that testing on vertebrate animals shall be undertaken only as a last resort.

The toxicokinetic screening studies also indicate that butyl paraben is not systemically bioavailable for a sufficiently long period of time to elicit DART or endocrine disrupting effects. This is supported by the recent findings from Hubbard et al. (2020). Finally, for the sodium salts of methyl paraben, ethyl paraben and propyl paraben, read-across of the NOAEL of 1000 mg/kg bw/day for repeated-dose toxicity and DART also appears justifiable on account of their very high similarities (*T*_c_ 0.98) as compared to the respective paraben.

To the best of the authors’ knowledge, this is the first time that a comprehensive dataset from higher-tier studies conducted following internationally agreed OECD TG test protocols and in full compliance with the GLP principles has become available for linear *n*-alkyl parabens. The data also enable a comprehensive evaluation of the endocrine disrupting potential of these parabens according to all five levels of the *OECD CF for Testing and Assessment of Endocrine Disrupting Properties* (OECD 2012). The higher-tier (OECD CF Level 4 and 5) studies on the shorter-chained linear *n*-alkyl parabens discussed here do not provide any indication that any endocrine activity (if it were present in vivo) would be sufficiently pronounced to overwhelm the physiological adaptive capacities of endocrine systems leading to adversity as indispensable prerequisite for endocrine disruption (WHO IPCS 2002).

As compared to the hazard-based approach pursued under the REACH Regulation for the assessment of endocrine disruptors, a risk-based approach is applied in other jurisdictions, such as USA, Canada, Australia and Japan (Brescia 2020). Risk-based approaches include exposure assessment in addition to hazard assessment to derive a conclusion on the safety of the respective substance. As highlighted by Brescia (2020), the application of a risk-based approach for the assessment of endocrine disruptors is scientifically justified since the available scientific evidence indicates that endocrine disruption exhibits a concentration threshold below which no effects will occur (Brescia 2020). Accordingly, the setting of concentration limits provides an additional safeguard to ensure consumer protection. For example, for all four shorter-chained linear *n*-alkyl parabens, EFSA (2004), SCCS (2013), and EMA (2015) have concluded that they are safe to the consumer when used in food, cosmetics and human medicinal products for oral use, respectively, provided that specific concentrations are not exceeded. This estimation has been re-confirmed by the findings from the present research article.

## Supplementary Information

Below is the link to the electronic supplementary material.

Supplementary file1 (DOCX 49 KB)
