# Supplementary material for: Application of grouping and read-across for the evaluation of parabens of different chain lengths with a particular focus on endocrine properties
Source: Arch Toxicol. 2021 Jan 18;95(3):853–81. doi: 10.1007/s00204-020-02967-0 (PMC7904550; doi:10.1007/s00204-020-02967-0)
Supplement: Supplementary file 1 — Supplementary file1 (DOCX 49 KB) [file 204_2020_2967_MOESM1_ESM.docx]

**Supplementary Information to:**

**Application of grouping and read-across for the evaluation of parabens of different chain lengths with a particular focus on endocrine properties**

Manuscript submitted to Archives of Toxicology

Authors:

*Susann Fayyaz^1^, Reinhard Kreiling^1*^, Ursula G. Sauer^2^*

1 Clariant Produkte (Deutschland) GmbH, Sulzbach, Germany

2 Scientific Consultancy – Animal Welfare, Neubiberg, Germany

* Corresponding author. Clariant Produkte (Deutschland) GmbH, Am Unisyspark 1, DE- 65843 Sulzbach, Germany. *Phone:* +49 6196 757 8781*; Fax:* +49 6196 757 8092. *E-mail address*: reinhard.kreiling@clariant.com (R. Kreiling).

**Supplementary Information SI-1**: Examples for findings in the extended one-generation reproductive toxicity studies (OECD TG 443) for propyl paraben that were assessed as non-toxicologically relevant

**Supplementary Information Table SI-2**: Physical and chemical properties of Na-methyl paraben, Na-ethyl paraben, Na-propyl paraben

**Supplementary Information Table SI-3**: Outcomes of acute toxicity, local toxicity and genotoxicity studies assessing Na-methyl paraben, Na-ethyl paraben, Na-propyl paraben

**Supplementary Information SI-4**: Commission (2017) scientific criteria for the determination of endocrine disrupting properties

**Supplementary Information SI-5**: Evaluation of butyl paraben under product-specific EU legislation and in the scientific literature

**Supplementary Information SI-1: Examples for findings in the extended one-generation reproductive toxicity studies (OECD TG 443) for propyl paraben that were assessed as non-toxicologically relevant**

1. Transient local reactions without toxicological relevance: Transient increased salivation (and moving the bedding) immediately after substance administration via oral gavage.

2. Effects that also occurred in the control animals or that were within the historical control range, e.g. in the female pups from parental females on postnatal day (PND) 0, minimal lower absolute and relative anogenital distance was observed in all dose groups as compared with the controls. Since these differences were only very slight and values were within the range of historical control data, they were assessed as not being toxicologically relevant.

3. Incidental findings, e.g. there were few marginal but statistically significant differences in clinical biochemistry parameters of male and female parental animals (i.e. alkaline phosphatase, total protein, urea, total bile acids, alanine aminotransferase, creatinine, potassium). As they were within the range of historical control data, not dose-dependent, not consistent between the genders and did not coincide with histopathological findings, these findings were not considered toxicologically relevant.

4. Slight effects that were assessed as non-toxicologically relevant: In parental females of the high dose group (and similarly, in the first-generation high-dose group males), slight but statistically significantly higher group mean thyroid stimulating hormone values were recorded. The thyroid stimulating hormone levels of few animals of this group were more prominently increased. Since the variability of the individual data was high and the findings were not associated with any microscopic finding of hypertrophy or an increased weight of the pituitary gland, they were assessed as not being toxicologically relevant.

**Table SI-2: Physical and chemical properties of Na-methyl paraben, Na-ethyl paraben, Na-propyl paraben**

| **Test substance** | **Na-methyl paraben** | **Na-ethyl paraben** | **Na-propyl paraben** |
| --- | --- | --- | --- |
| **CAS number** | **5026-62-0** | **35285-68-8** | **35285-69-9** |
| **Chemical structure** | 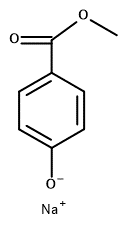 | 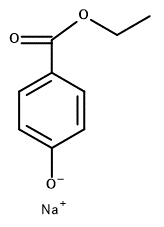 | 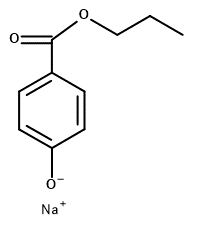 |
| **IUPAC name** | Sodium methyl 4-HBA | Sodium ethyl 4-HBA | Sodium propyl 4-HBA |
| **Purity** | 95-98 % | 95-100 % | >95 % |
| **Impurities** | 1-3 % 4-HBA; 1-2 % sum of unknowns | < 3 % 4-HBA; < 2 % sum of unknowns | < 4 % HBA; < 2 % sum of unknowns |
| **Appearance** | White crystalline solid | White solid | Colourless crystals or white powder, solid |
| **MW (g/mol)** | 174.13 | 188.16 | 202.8 |
| **Melting point at 1013 hPa [°C]** | 313 (OECD TG 102) | 268 (OECD TG 102) | 302 (OECD TG, 102) |
| **pK_a_** | 8.4 (OECD TG 112) | 8.4 (WoE) | 8.46 (WoE) |
| **Log K_ow_** | -0.63 at 20 °C, pH 10  (OECD TG 107) | -0.14 at 23 °C, pH 10.4  (OECD TG 107) | 0.27 at 23 °C, pH 10.8  (OECD TG 107) |
| **Water solubility**  **[g/L]** | 418 at 20 °C, pH 11.4  (OECD TG 105) | > 1000 at 23 °C  (OECD TG 105) | > 1000 at 20 ± 0.5 °C  (OECD TG 105, v1995) |
| **T_c_ [a]** | 0.98 as compared to  methyl paraben | 0.98 as compared to  ethyl paraben | 0.98 as compared  propyl paraben |

Footnote to Table SI-2:

Abbreviations: 4-HBA: 4-Hydroxybenzoate; CAS: Chemical Abstract Service; IUPAC: International Union of Pure and Applied Chemistry; Log K_ow_: Octanol-water partition coefficient; MW: Molecular weight; PK_a_: (Negative base-10 logarithm of) acid dissociation constant; T_c_: Tanimoto similarity coefficient; WoE: Weight-of-evidence.

This table contains data that are freely available on the ECHA dissemination portal (<https://.echa.europa.eu>). Purity of all test items was confirmed via Certificate of Analysis by the Quality Assurance Unit of the authors’ laboratories. Generally, the measurement of physico-chemical properties was performed or commissioned by Clariant Produkte (Deutschland) GmbH. WoE indicates that the respective properties were established by WoE evaluation of all available published data.

[a] The Tanimoto similarity coefficient (Tc) was calculated using the Open Babel open source chemistry toolbox, version 2.4.1. A T_c_ > 0.85 is assessed as indicating high similarity (see Table 3 in paper for further details).

**Table SI-3: Outcomes of acute toxicity, local toxicity and genotoxicity studies assessing Na-methyl paraben, Na-ethyl paraben, Na-propyl paraben**

| **Endpoint** | **Na-Methyl paraben** | **Na-Ethyl paraben** | **Na-Propyl paraben** |
| --- | --- | --- | --- |
| **CAS Number** | **5026-62-0** | **35285-68-8** | **35285-69-9** |
| **Acute oral toxicity studies using rats: LD_50_ [mg/kg bw] (in brackets: OECD TG and date of study)** | | | |
| **Acute toxicity** | **> 5000** (OECD 401; 1982) | **3100**  OECD 401; 1982) | **> 5000**  OECD 401; 1982) |
| **Local toxicity studies using rats: Classification (in brackets: OECD TG and date of study)** | | | |
| **Skin irritation / corrosion** | **Skin irritating** [a]  (OECD 439; 2012) | **Not irritating**  (OECD 404; 1981) | **Not irritating**  (OECD 404; 1982) |
| **Eye irritation / corrosion** | **Serious eye damage** (CFR, Title 16, 1500.42) | **Irritating**  (OECD 405; 1982) | **Serious eye damage** (OECD 405; 1983) |
| **Skin**  **sensitisation** | **Not sensitising**  (read-across  from methyl paraben) | **Not sensitising**  (read-across  from ethyl paraben) | **Not sensitising**  (read-across  from propyl paraben) |
| ***In vitro* and *in vivo* genotoxicity studies: Classification (in brackets: OECD TG and date of study)** | | | |
| **Mutagenicity  in bacteria** | **Not mutagenic** [a]  (OECD 471; 2012) | **Not mutagenic**  (read-across from ethyl paraben) | **Not mutagenic**  (read-across from propyl paraben) |
| **Mutagenicity  in mammalian cells** | **Not mutagenic** [a]  (OECD 476; 2012) | Not a REACH information requirement for this tonnage  (1-10 tonnes / year) | Not a REACH information requirement for this tonnage  (1-10 tonnes / year) |
| **Genetic toxicity *in vivo*** | Not a REACH information requirement for this tonnage  (10-100 tonnes / year) |  |  |

Footnote to Table SI-3:

Abbreviations: CFR: Code of Federal Regulations.

This table contains data that are freely available on the ECHA dissemination portal (<https://.echa.europa.eu>). Years relate to the date of study performance; the respective most recent version of the respective OECD TG was applied.

The classification for local toxicity and genotoxicity was performed in accordance with the *Globally Harmonized System of Classification and Labelling of Chemicals* that has been implemented in EU *Regulation (EC) 1272/2008 on classification, labelling and packaging of substances and mixtures* (see paper for references).

[a] Study commissioned by Clariant Produkte (Deutschland) GmbH.

**Supplementary Information SI-4:** **Commission (2017) scientific criteria for the determination of endocrine disrupting properties (similarly in Commission (2018); emphasis by the authors of the present article)**

*“A substance shall be considered as having endocrine-disrupting properties that may cause adverse effect in humans if […it…] meets all of the following criteria, unless there is evidence demonstrating that the adverse effects identified are not relevant to humans:*

*1. it shows an adverse effect in an intact organism or its progeny, which is a change in the morphology, physiology, growth, development, reproduction or life span of an organism, system or (sub)population that results in an impairment of functional capacity, an impairment of the capacity to compensate for additional stress or an increase in susceptibility to other influences;*

*2. it has an endocrine mode of action*, i.e. it alters the function(s) of the endocrine system;*

*3. the adverse effect is a consequence of the endocrine mode of action*.”*

* In bullet 2, the term ‘endocrine mode of action’ should be understood as ‘endocrine activity’ (i.e. *in vitro* and/or *in vivo* mechanistic information) and in bullet 3 as ‘mode of action postulated as link between endocrine activity and adverse effect’ (EFSA and ECHA 2018).

**References to SI-4**

Commission (2017) Commission Delegated Regulation (EU) 2017/2100 of 4 September 2017 setting out scientific criteria for the determination of endocrine-disrupting properties pursuant to Regulation (EU) No 528/2012 of the European Parliament and Council. OJ EU L 301(60):1-12.

Commission (2018) Commission Regulation (EU) 2018/605 of 19 April 2018 amending Annex II to Regulation (EC) No 1107/2009 by setting out criteria for the determination of endocrine disrupting properties. OJ EU L 101:33–36.

EFSA, ECHA (2018) European Food Safety Authority and European Chemicals Agency with the technical support of the Joint Research Centre (Andersson N, Arena M, Auteri D, Barmaz S, Grignard E, Kienzler A, Lepper P, Lostia AM, Munn S, Parra Morte JM, Pellizzato F, Tarazona J, Terron A, Van der Linden S). Guidance for the identification of endocrine disruptors in the context of Regulations (EU) No 528/2012 and (EC) No 1107/2009. ECHA-18-G-01-EN; EFSA J 16:1661–170.

**SI-5: Evaluation of butyl paraben under product-specific EU legislation and in the scientific literature**

In 2010, the SCCS conducted the risk assessment for butyl paraben *“using the very low NOEL value of 2 mg/kg bw/day derived from a study where juvenile rats were exposed after subcutaneous administration of 2 mg butylparaben/kg/day for 17 days (postnatal days 2-18; (Fisher et al. 1999) …”* (SCCS, 2010). By contrast, the U.S.-based Cosmetics Ingredient Review Expert Panel (CIR, 2019) considered that the study by Fisher et al. (1999) suffered from critical limitations including not being an OECD TG study, testing only one postpartum dose, subcutaneous substance administration, and excluding important DART endpoints. Considering that only one single dose was tested, that moreover by far undercuts the limit dose of 1000 mg/kg bw/day, a NOEL of 2 mg/kg bw/day does not preclude that effects might also not be elicited at higher concentrations up until the limit dose. Therefore, it is an unacceptable and invalid approach to select such a NOEL as point-of-departure for the safety assessment (ECHA, 2012).

In February 2020, the Danish Environmental Protection Agency (DK EPA, 2020) submitted a proposal for identifying butyl paraben as substance of very high concern pursuant to REACH Article 57. The main adverse effect purported by DK EPA (2020) was irreducible reduced sperm count and sperm quality observed in rodent studies addressing perinatal substance exposure. In such studies, test items are administered to pregnant dams from implantation (gestational day (GD) 6-7) up until the end of lactation (postnatal day (PND) 21-22). DK EPA (2020) further stated that there was supportive evidence for the evolvement of such effects following pubertal and/or adult exposure, while conceding that there were some inconsistencies between these studies. Table SI-5 provides an overview of the key studies cited in DK EPA (2020) to support the concern for reduced sperm count and sperm quality (i.e. Kang et al., 2002; Zhang et al., 2014; Boberg et al., 2016; Guerra et al., 2017).

**Table SI-5: Key studies cited in DK EPA (2020) to support the concern that butyl paraben might elicit reduced sperm count and sperm quality**

| **Study** | **Rat strain** | **Dose groups  (mg/kg bw/day)** | **Route of administration (to the dams)** | **Exposure duration** |
| --- | --- | --- | --- | --- |
| Kang et al. (2002) | Sprague-Dawley | 100, 200 | Subcutaneous injection | GD 6 to PND 20 |
| Zhang et al. (2014) | Wistar | 64, 160, 400, 1000 | Oral gavage | GD 7 to PND 21 |
| Boberg et al. (2016) | Wistar | 10, 100, 500 | Oral gavage | GD 7 to PND 22 |
| Guerra et al. (2017) | Wistar | 10, 100, 200 | Subcutaneous injection | GD 12 to PND 21 |

An in-depth evaluation of the studies cited in DK EPA (2020) would exceed the scope of the present article. Nonetheless, it is noteworthy that the subcutaneous route of administration applied by Kang et al. (2002) and Guerra et al. (2017) is generally an irrelevant exposure route for the intended uses of parabens (CIR, 2019). As regards the Boberg et al. (2016) study, CIR (2019) highlighted the following limitations:

1. The reduction of epididymal sperm count showed the same effect across all doses, whereas a dose-response relationship is expected between exposure to oestrogen active substances and sperm count decrease.
2. Wide variation exists in measuring epididymal sperm count between different laboratories and/or different experimental technicians. Thus, a decline in sperm counts warrants further validation.
3. The data for DART endpoints did not show consistency from 10-100 mg/kg bw/day when compared to other DART studies with similar butyl paraben exposure (CIR, 2019).

Zhang et al. (2014) recorded dose-dependently reduced epididymal sperm count and daily sperm production upon oral gavage exposure to 400 and 1000 mg butyl paraben/kg bw/day. Further, Zhang and co-workers reported altered serum hormone levels at different postnatal timepoints and reduced anogenital distance at PND 1 and 4 and delayed preputial separation in the male offspring. The biological relevance of the observation that anogenital distance was reduced cannot be established since Zhang et al. (2014) did not relate this parameter to the pups’ body weight or to a historical control range. Reduced anogenital distance should not *per se* be considered an adverse effect (Schwartz et al., 2019). Also, comparable findings were not reported by Taxvig et al. (2008), exposing Wistar rat dams from GD 7 - GD 21 even via subcutaneous administration to 200 or 400 mg butylparaben mg/kg bw/day. Comparable findings were also not reported by Boberg et al. (2008), exposing pregnant Wistar rats from GD 7 – GD 21 by oral gavage to 1-600 mg butylparaben mg/kg bw/day; even though the latter is erroneously contested in Boberg et al. (2016). Similarly, Hoberman et al. (2008) reported no effects caused by butyl paraben in a feeding study using male rats (exposure from PND 22-78) up to the limit dose (10,000 ppm; corresponding to 1088 ± 68 mg/kg bw/day).

Notably, CIR (2019) used the findings by Zhang et al. (2014) to derive a NOAEL of 160 mg/kg bw/day as point-of-departure to establish concentration limits for the safe use of butyl paraben.

However, recent evidence from a two-generation reproductive assessment rat feeding study conducted within the U.S. National Toxicology Program (Hubbard et al., 2020) showed no association between oral exposure to 5,000 ppm, 15,000 ppm and 40,000 ppm butyl paraben and adverse alterations of fertility, fecundity, pubertal attainment or any reproductive parameters in the parent, first, or second generation (but exposure-dependent increases in liver weight and incidences of non-neoplastic liver lesions). Notably, in this study, the mid-dose (15,000 ppm) generally exceeded the OECD limit dose of 1000 mg/kg bw/day by up to two-fold, whereas the high-dose group (40,000 ppm) exceeded it by at least 2.5-fold and up to 6.7-fold (depending on the animals’ life cycle stage, e.g. gestation, lactation). Hence, the findings from this recent two-generation study (Hubbard et al., 2020) support the conclusion drawn in the present research article, that, for butyl paraben extrapolation of a NOAEL of 1000 mg/kg bw/day for repeated-dose toxicity and DART appears justifiable and that butyl paraben further does not exhibit any endocrine disrupting properties.

**References to Supplementary Information SI-5**

Boberg J, Axelstad M, Svingen T, Mandrup K, Christiansen S, Vinggaard AM, Hass U. 2016. Multiple endocrine disrupting effects in rats perinatally exposed to butylparaben. Toxicol.Sci. 152(1):244-256.

Boberg J, Metzdorff S, Wortziger R, Axelstad M, Brokken L, Vinggaard AM, Dalgaard M, Nellemann C. 2008. Impact of diisobutyl phthalate and other PPAR agonists on steroidogenesis and plasma insulin and leptin levels in fetal rats. Toxicology. 250(2-3):75-81.

Chapin RE, Sloane RA. 1997. Reproductive Assessment by Continuous Breeding: Evolving study design and summaries of ninety studies. Env Health Perspect. 105(Suppl 1):199-205.

CIR. 2019. Cosmetic Ingredient Review Expert Panel. Amended safety assessment of parabens as used in cosmetics. Final report; release date: 7 October 2019; Cosmetic Ingredient Review, Washington DC, USA.

DK EPA. 2020. Danish Environmental Protection Agency. Annex XV report. Proposal for identification of a substance of very high concern on the basis of the criteria set out in REACH Article 57. Substance name: Butyl 4-hydroxybenzoate (butylparaben); EC number: 202-318-7; CAS number. 94-26-8; February 2020.

ECHA. 2012. Guidance on information requirements and chemical safety assessment. Chapter R.8. Characterisation of dose [concentration]-response for human health. Version 2.1; ECHA-2010-G-19-EN; November 2012.

EFSA, ECHA. 2018. European Food Safety Authority and European Chemicals Agency with the technical support of the Joint Research Centre (Andersson N, Arena M, Auteri D, Barmaz S, Grignard E, Kienzler A, Lepper P, Lostia AM, Munn S, Parra Morte JM, Pellizzato F, Tarazona J, Terron A, Van der Linden S). Guidance for the identification of endocrine disruptors in the context of Regulations (EU) No 528/2012 and (EC) No 1107/2009. ECHA-18-G-01-EN; EFSA J 16:1661–170.

Fisher J, Turner K, Brown D, Sharpe R. 1999. Effect of neonatal exposure to estrogenic compounds on development of the excurrent ducts of the rat testis through puberty to adulthood. Environ Health Perspect. 107(5):397-405.

Guerra MT, Sanabria M, Leite GA, Borges CS, Cucielo MS, Anselmo-Franci JA, Foster WG, Kempinas WG. 2017. Maternal exposure to butyl paraben impairs testicular structure and sperm quality on male rats. Environ Toxicol. 32(4):1273-1289.

Hoberman AM, Schreur DK, Leazer T, Daston GP, Carthew P, Re T, Loretz L, and Mann P. 2008. Lack of effect of butylparaben and methylparaben on the reproductive system in male rats. Birth Defects Res. B Dev. Reprod. Toxicol. 83(2):123-133.

Hubbard TD, Brix A, Blystone CR, McIntyre BS, Shockley K, Cunny H, Waidyanatha S, Turner KJ, McBride S, Roberts GK. 2020. Butylparaben multigenerational reproductive assessment by continuous breeding in Hsd:Sprague Dawley SD rats following dietary exposure. Reprod Toxicol. 96:258-272.

Kang S, Che JH, Ryu DY, Kim TW, Li GX, Lee YS. 2002. Decreased sperm number and motile activity on the F_1_ offspring maternally exposed to butyl p-hydroxybenzoic acid (butyl paraben). Sci Total Environ. 461-462:214-221.

SCCS. 2010. European Commission Directorate General for Health and Consumers. Scientific Committee on Consumer Safety. Opinion on parabens COLIPA No. P82; adopted at the 9^th^ plenary on 14 December 2010. SCCS/1348/10; revision 22 March 2011.

Schwartz CL, Christiansen S, Vinggaard AM, Axelstad M, Hass U, Svingen T. 2019. Anogenital distance as a toxicological or clinical marker for fetal androgen action and risk for reproductive disorders. Arch Toxicol. 93(2):253-272.

Taxvig C, Vinggaard AM, Hass U, Axelstad M, Boberg J, Hansen PR, Frederiksen H, Nellemann C. 2008. Do parabens have the ability to interfere with steroidogenesis? Toxicol Sci. 106(1):206-13.

Zhang L, Dong L, Ding S, Qiao P, Wang C, Zhang M, Zhang L, Du Q, Li Y, Tang N, Chang B. 2014. Effects of n-butylparaben on steroidogenesis and spermatogenesis through changed E(2) levels in male rat offspring. Environ. Toxicol. Pharmacol. 37(2):705-717.
